# Dual benefits of neuromuscular training in adolescent volleyball players: knee injury prevention and athletic performance share a common mechanistic foundation: a structured narrative review

**DOI:** 10.3389/fspor.2026.1849255

**Published:** 2026-06-24

**Authors:** Shizhao Chen, Qingzi Liu, Zihui Hong

**Affiliations:** 1 School of Strength and Conditioning, Beijing Sport University, Beijing, China; 2 St. Michaels University School, Victoria, BC, Canada; 3 Faculty of Business and Economics, Monash University, Clayton, VIC, Australia

**Keywords:** adolescent athletes, anterior cruciate ligament (ACL), athletic performance, biomechanics, neuromuscular training, peak height velocity (PHV), volleyball

## Abstract

Adolescent volleyball players face elevated knee injury risk. Anterior cruciate ligament (ACL) injuries and patellar tendinopathy are the two dominant conditions. Approximately 62% of ACL injuries occur during spike landings. During peak height velocity (PHV), patellar tendon strain reaches 7.6%–8.5%, approaching the microstructural damage threshold of 9.0%. This review evaluated whether neuromuscular training (NMT) reduces biomechanical injury risk and improves athletic performance. Multimodal NMT reduced overall injury rates by 40% and halved ACL injury risk (OR=0.54). The protective effect was strongest in females aged 14–18 (OR=0.28) and absent above age 18. NMT also improved vertical jump height (Cohen's d = 0.82) and dynamic stability (SMD = 0.63). Female players showed greater jump gains than males (ES = 1.3 vs. 0.5). Injury prevention and performance benefits share the same mechanistic foundation. Optimal dosing is 15–20 min per session, two to three times per week, for at least eight weeks. Maturity-stratified load management is essential for circa-PHV athletes. Volleyball-specific, maturity-indexed RCTs are needed to confirm these findings.

## 1 Introduction

### 1.1 Biomechanical demands of volleyball

Volleyball places high mechanical demands on the lower extremities. The key actions (including spiking, blocking, and defensive coverage) involve repeated explosive jumps, forceful landings, and rapid changes of direction. In field sports, these intense movements are interspersed with slower activity. In volleyball, they occur back to back within each rally.

**Figure 1:**
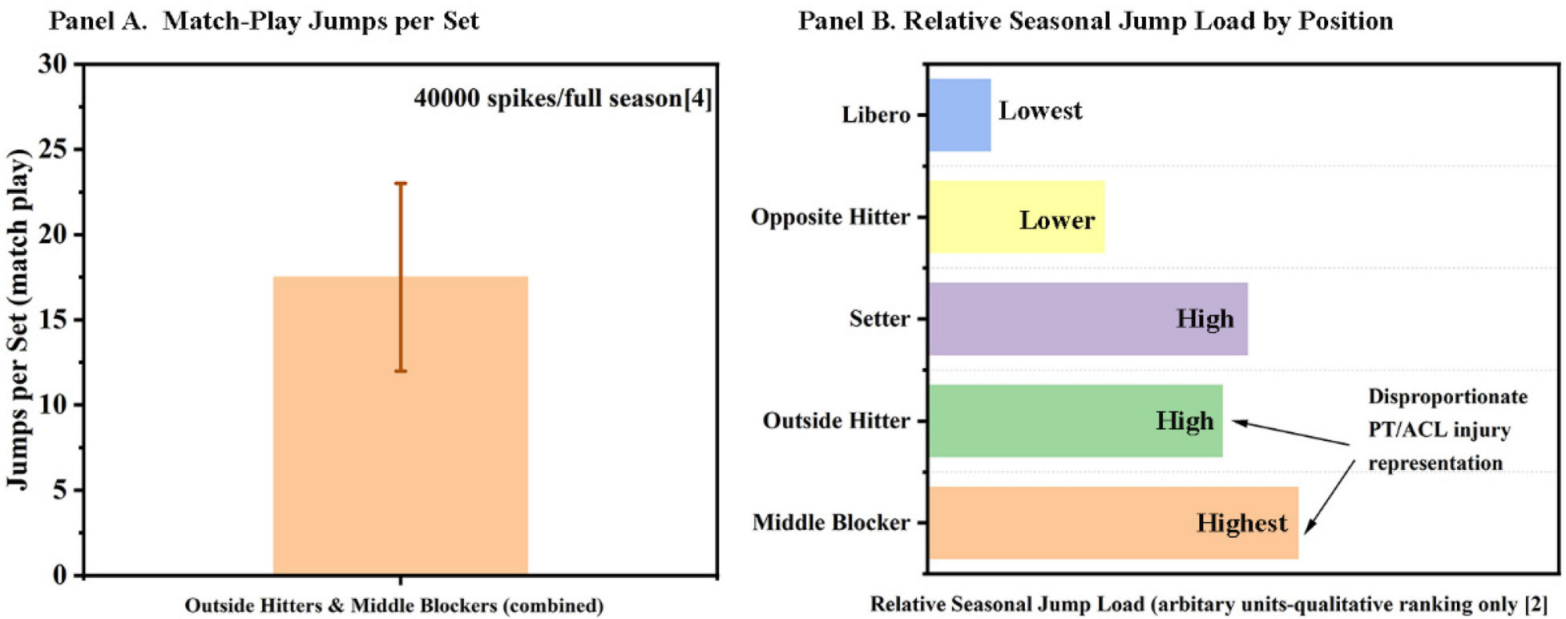
Jump exposure and positional load distribution in competitive volleyball.

Jump volume is a common measure of physical load in volleyball. Bahr et al. studied elite players aged 16–18 and recorded 11,943 jumps across one week of training and ten official matches. Jump counts varied considerably between teammates. Higher total jump volume was associated with greater risk of patellar tendinopathy (1). At the professional level, Skazalski et al. followed 14 players over a full competitive season of 142 sessions. The team completed 129,173 jumps in total, averaging approximately 910 jumps per session (2). Setters and middle blockers carried the highest individual loads. Weekly jump counts also fluctuated substantially, some players doubled their volume from one week to the next (2). During matches, outside hitters and middle blockers perform 12–23 jumps per set (3). Over a full season, elite players may spike around 40,000 times. Each spike represents a discrete loading event for the knee extensor muscles (Figure 1) (4).

Multi-directional movement adds further injury risk. During matches, players must react quickly to unpredictable ball trajectories. This demands frequent lateral shuffles, diagonal approach steps, and sudden stops. In unexpected blocking situations, vertical ground reaction forces increase, hip extension moments rise, and knee flexion power decreases. This combination reduces the body's capacity to absorb shock precisely when loads are greatest (5). Movements in the non-dominant direction produce larger knee valgus moments than those in the dominant direction. Lateral and asymmetric landings therefore represent a distinct, higher-risk category (5). Sex differences also contribute to biomechanical risk. Female players exhibit different movement patterns and force profiles during the spike approach and landing compared to male players, meaning injury risk is not uniform across all athletes (6).

Middle blockers and outside hitters carry the highest jump loads over a season. They also show up more often in patellar tendinopathy statistics (1, 2). For teenage players, the neuromuscular system is still developing. Although overall injury rates in adolescent volleyball players are lower than in adults (7), injuries sustained during this developmental window carry disproportionately severe long-term consequences: ACL injury prevalence is higher at high school than at collegiate level (8), and youth-onset patellar tendinopathy has been associated with competitive retirement in one in five affected players (9).

### 1.2 The adolescent vulnerability window

Adolescent volleyball players are not simply smaller versions of adult players. Puberty creates a period of temporary vulnerability in the musculoskeletal system. During peak height velocity (PHV), bones grow faster than the surrounding muscles and tendons can adapt. Limb inertia increases, the center of mass shifts upward, and body mass rises. Muscle strength and stiffness do not increase at the same rate (10, 11). Girls typically reach PHV at around age 12, growing up to 9 cm per year. Boys reach PHV approximately two years later, growing up to 10.3 cm per year (10). The mechanical consequences are substantial. At age 14, maintaining knee extension in a seated position requires approximately 4.7 times more torque than the same task at age six (11).

Injury rates follow maturation stage more closely than chronological age. A scoping review of elite youth athletes found that injury burden increased steadily with maturation (12). Growth-related injuries peaked around the time of PHV (12). Rapid gains in limb length and body mass near PHV were independently associated with higher rates of overuse and non-contact injuries. This relationship held after accounting for total training exposure (12).

Puberty follows different trajectories in girls and boys, producing distinct injury risk profiles. In girls, pelvic widening increases the Q-angle and creates a natural valgus tendency at the knee (10). Neuromuscular adaptation does not keep pace with skeletal growth during this period (Figure 3). In boys, testosterone drives a concurrent neuromuscular growth phase. Muscle mass and motor coordination improve together, supporting control of larger and heavier limbs (13). Girls do not receive the same hormonal stimulus. Their neuromuscular gains are small relative to the magnitude of skeletal change (13). Hewett et al. tracked 181 athletes and measured this difference directly. Post-pubertal girls showed substantially higher knee abduction moments during drop-landing than pre-pubertal girls. This pattern was absent in boys (14, 15).

Skeletal growth, insufficient neuromuscular adaptation, and sex-specific hormonal responses converge during adolescence. Together, these factors place adolescent female volleyball players in a high-risk group. The physical demands of volleyball coincide with the developmental window in which their bodies are least equipped to tolerate load.

### 1.3 Research Gap and study objectives

The NMT evidence base in youth sports is substantial but unevenly distributed. Most high-quality RCTs have been conducted in football. FIFA 11 + reduces overall injury rates by 39% and forms the backbone of international guidelines (16, 17). Evidence from basketball (18) and handball (19) has grown over the past decade. Volleyball-specific evidence remains sparse. A 2025 systematic review identified only seven eligible RCTs, further limited by heterogeneous samples, single-component designs, and absent age stratification (20).

This gap has two important consequences. First, volleyball imposes a biomechanical loading profile that is structurally distinct from football or basketball. Single-leg spike landings, high positional jump accumulation, and frequent unanticipated lateral movements define a risk environment that generic multi-sport programs are not designed to target (21). Evidence-based ACL prevention guidelines acknowledge that volleyball-specific asymmetric landing patterns require sport-tailored intervention beyond current standard protocols (8). Second, the adolescent developmental window is precisely the period when NMT produces its greatest protective effect (15). Yet most volleyball research has studied adult cohorts or ignored maturation status. The combination of a sport-specific mechanical environment and a developmentally sensitive population has not been addressed as a unified question.

A further gap concerns the prevention–performance relationship. NMT is routinely framed as a prevention tool, limiting coach uptake (22). Multi-sport data indicate NMT can simultaneously improve vertical jump height (23), agility (24) and dynamic stability (20), but whether these gains occur in a volleyball-specific adolescent context, and whether prevention and performance adaptations share mechanisms, remains unexamined.

This review addresses both gaps through two research questions. RQ1: Does NMT effectively reduce biomechanical risk factors for knee injury in adolescent volleyball players, including dynamic knee valgus, reduced knee flexion at landing, and impaired neuromuscular control during sport-specific tasks? RQ2: Does the same NMT intervention simultaneously produce quantifiable improvements in athletic performance in this population? Both questions are examined within a single analytical framework, spanning the biomechanical and performance dimensions of the available evidence.

## 2 Methods

### 2.1 Search strategy and databases

A systematic literature search was conducted in six electronic databases: PubMed/MEDLINE, SPORTDiscus, Web of Science, Scopus, the Cochrane Library, and EMBASE. All searches were performed in June 2025. The search covered publications from January 2000 to March 2026. The year 2000 was selected as the lower boundary to ensure methodological consistency, as standardised biomechanical measurement protocols and neuromuscular training terminology were not uniformly applied in the volleyball and sports injury literature prior to this date.

**Figure 2:**
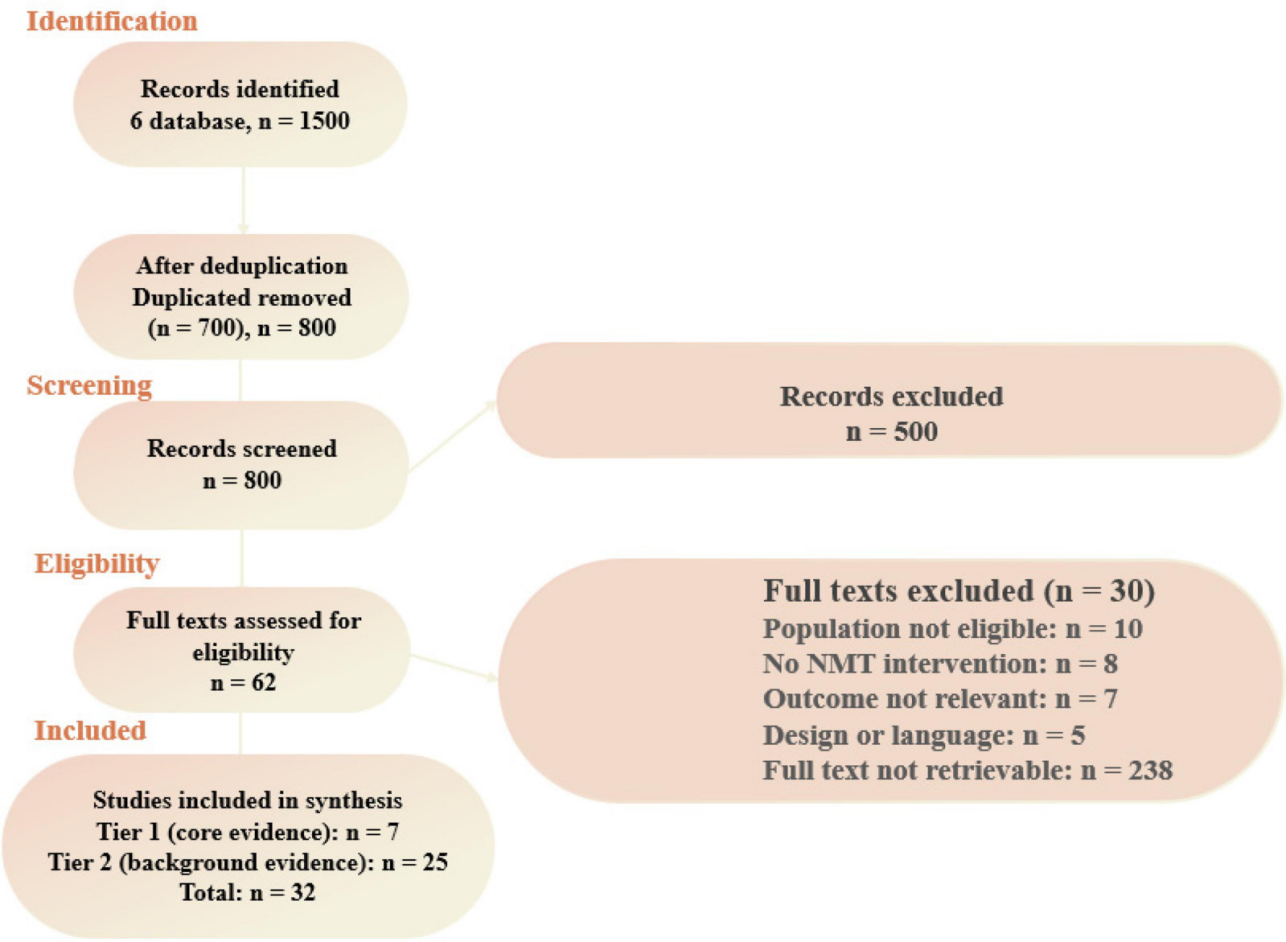
PRISMA flow diagram illustrating the literature search and study selection process.

This review is a structured narrative review. It differs from a traditional narrative review in that it employs a reproducible search strategy and formal quality appraisal. It differs from a systematic review in that it does not perform exhaustive search coverage, dual-independent screening, or risk-of-bias pooling. This intermediate design is consistent with current recommendations for improving narrative review rigor (21). The PRISMA flow diagram (Figure 2) is included for transparency, not to assert systematic review status (22). Quality appraisal using PEDro and AMSTAR-2 assigns evidence confidence grades used throughout Sections 3–5. No GRADE profiling or sensitivity analysis was conducted. The two-tier evidence classification distinguishes primary from contextual evidence. It reflects a structured interpretive hierarchy, not a claim to systematic coverage.

Search terms were organized into three concept blocks and combined using Boolean operators. The first block targeted the study population: (“volleyball” OR “volleyball player” OR “volleyball athlete”). The second block targeted the intervention: (“neuromuscular training” OR “NMT” OR “injury prevention program” OR “plyometric training” OR “proprioceptive training” OR “core stability training” OR “balance training” OR “landing biomechanics training”). The third block targeted the outcome domains: (“knee injury” OR “ACL” OR “anterior cruciate ligament” OR “patellar tendinopathy” OR “jumper's knee” OR “knee valgus” OR “landing mechanics” OR “vertical jump” OR “agility” OR “dynamic stability”). Terms within each block were combined with OR; the three blocks were combined with AND. Subject headings (MeSH in PubMed, DE terms in SPORTDiscus) were used alongside free-text terms to maximize retrieval sensitivity.

All records were screened by the lead author based on titles and abstracts. Full texts were retrieved for all potentially eligible records. A second reviewer independently verified a random 20% sample of screened records to confirm screening consistency.

Included literature was classified into two tiers. Tier 1 comprised direct volleyball-specific evidence: studies with adolescent volleyball players as the primary population, reporting biomechanical or performance outcomes of NMT. These form the primary evidentiary basis for Sections 3–5. Tier 2 comprised indirect background evidence: studies in adult volleyball populations or other sports (football, basketball, handball), used to establish mechanistic and epidemiological context. Findings from all volleyball-specific studies are marked (VS) throughout Sections 3–6. Findings without this tag represent mechanistic extrapolations from non-volleyball populations and are explicitly identified as such. Tier 2 sources are identified as such throughout the text and were not used to draw primary conclusions about the adolescent volleyball population. The search was not restricted by language; however, only records with full text available in English or Chinese were included in the final synthesis. Where Tier 1 evidence is absent for a specific outcome, Tier 2 evidence is used to establish mechanistic plausibility and directional consistency. In such cases, conclusions are graded as limited or moderate evidence and are not treated as directly applicable to the adolescent volleyball population without qualification.

To make the evidentiary basis of each conclusion explicit, a standardised inline tag (VS) is used throughout Sections 3–6. This tag appears immediately after any finding drawn directly from a volleyball-specific study, whether Tier 1 or Tier 2. Findings without this tag are based on multi-sport or non-volleyball evidence and represent mechanistic extrapolations. The (VS) tag is used alongside the existing evidence confidence grades, so that readers can assess both source specificity and methodological quality at each point.

### 2.2 Inclusion and exclusion criteria

Included literature was classified according to the two-tier framework described in Section 2.1. Separate criteria were applied to each tier.

Tier 1 studies were required to meet all of the following criteria. The primary study population comprised adolescent athletes, defined as individuals aged 10–18 years engaged in volleyball training or competition. The intervention involved at least one component of neuromuscular training, including but not limited to plyometric training, proprioceptive training, balance training, core stability training, eccentric resistance training, or structured landing mechanics correction. At least one outcome relevant to knee injury risk or athletic performance was reported. Eligible outcomes included dynamic knee valgus, knee flexion angle at landing, vertical ground reaction force, neuromuscular activation timing, vertical jump height, dynamic stability, or agility. Study designs eligible for Tier 1 inclusion were randomized controlled trials, quasi-randomized controlled trials, and controlled trials with a comparator group. Systematic reviews and meta-analyses with adolescent volleyball players as the primary population were also eligible.

Tier 2 studies were included where they met the following criteria. The study population comprised adult volleyball players, or athletes in other pivoting or jumping sports including football, basketball, and handball. The study reported mechanistic, epidemiological, or dose-response evidence relevant to the research questions of this review. Study designs included randomized controlled trials, systematic reviews, meta-analyses, prospective cohort studies, and cross-sectional biomechanical studies.

The following were applied as exclusion criteria across both tiers. Studies published before January 2000 were excluded on the grounds of methodological inconsistency in biomechanical measurement and neuromuscular training terminology prior to this date. Case reports, editorials, conference abstracts, and studies for which full text was unavailable in English or Chinese were excluded. Studies that did not report extractable outcome data relevant to knee injury risk or athletic performance were excluded. Studies in which the primary population were non-athletic or clinical populations with existing knee pathology were excluded from both tiers.

Where a study population included both adolescent and adult athletes without separate reporting by age group, the study was classified as Tier 2 and used for background context only.

### 2.3 Quality assessment

The methodological quality of included Tier 1 studies was assessed using two validated tools, selected according to study design.

For randomized and quasi-randomized controlled trials, the Physiotherapy Evidence Database (PEDro) scale was applied. The PEDro scale comprises 11 criteria, of which 10 are scored to produce a total out of 10. Following Maher et al. (25), scores of 9–10 were classified as excellent quality; 6–8 as good quality; 4–5 as fair quality; and 3 or below as poor quality. The scale evaluates random allocation, concealed allocation, baseline comparability, blinding of participants and therapists, blinding of assessors, completeness of follow-up, intention-to-treat analysis, between-group statistical comparisons, and reporting of point estimates with variability measures.

For included systematic reviews and meta-analyses, quality was assessed using AMSTAR-2 (26). AMSTAR-2 contains 16 items, seven of which are designated as critical domains (items 2, 4, 7, 9, 11, 13, and 15). These concern: PICO question specification, literature search comprehensiveness, study selection in duplicate, risk of bias assessment, use of appropriate meta-analytic methods, consideration of risk of bias when interpreting results, and assessment of publication bias. An overall confidence rating (High, Moderate, Low, or Critically Low) is assigned based on the number and location of weaknesses across critical and non-critical items.

Quality assessment was conducted independently by two authors. Discrepancies were resolved by discussion until consensus was reached. Quality ratings were not used as exclusion criteria. Low-quality studies were retained in the synthesis but are explicitly identified as such in the text. Three evidence confidence levels are applied throughout: high (multiple good-to-excellent quality studies with consistent findings); moderate (single high-quality study or multiple fair-quality studies); and low (poor-quality, sparse, inconsistent, or exclusively Tier 2 evidence). These are indicated parenthetically as (strong evidence), (moderate evidence), and (limited evidence) respectively, ensuring conclusions are weighted according to underlying methodological quality.

Heterogeneity across included meta-analyses was evaluated using the I^2^ statistic. Established thresholds were applied following Higgins and Thompson (27): 0%–25% indicates low heterogeneity, 25%–50% moderate, 50%–75% substantial, and above 75% considerable. Where substantial or considerable heterogeneity was identified, pooled estimates are interpreted with additional caution. Heterogeneity findings for specific outcomes are discussed in Sections 5.1 and 5.2.

All effect sizes, standardized mean differences, odds ratios, and I^2^ statistics reported in this review are extracted directly from the cited meta-analyses. No independent pooling, weighting, or heterogeneity testing was conducted by the authors. These values are reported to support direct evaluation of source evidence precision and consistency. They also underpin the evidence confidence grading system applied throughout Sections 3–5. This practice is consistent with structured narrative review methodology (22).

PEDro and AMSTAR-2 were applied only to the three Tier 1 studies. They were not applied to Tier 2 sources. Their purpose was to assign evidence confidence grades used in Sections 3–5. No risk-of-bias pooling or GRADE profiling was performed. PEDro was selected as the validated instrument for controlled trials in exercise research (25). AMSTAR-2 was selected as the current standard for appraising systematic reviews and meta-analyses (26). This scope-limited application is consistent with structured narrative review methodology (22).

## 3 Epidemiology and biomechanics of knee injuries in youth volleyball

### 3.1 Injury patterns and prevalence in youth volleyball

Volleyball injury profile is dominated by the lower limb. In adult populations, lower limb injuries account for 50%–60% of all injuries, with ankle sprains the most frequent acute event and patellar tendinopathy the dominant overuse condition (5). In youth populations, absolute rates are lower but the structural pattern is consistent and long-term consequences are disproportionate.

A 2023 review of athletes aged 12–18 years (five cohort studies; *n* = 3698, predominantly female) found injury incidence ranging from 1.51/1,000 player hours to 12.4/10,000 AEs, with an overall prevalence of 1.6 ± 1.7 per 100 AEs (7). The ankle, distal upper limb, and knee were the highest-injury sites, in that order. Female youth volleyball athletes showed the lowest acute and overuse injury rates compared to eight other junior high school sports. Within the youth volleyball population, however, older athletes consistently showed higher injury rates (7). Within the youth volleyball population, however, older athletes consistently showed higher injury rates (7) (VS).

A 2025 systematic review (15 cohort studies; *n* = 3,313,248 athletes) identified non-contact mechanisms in 85%–97% of volleyball ACL injuries, with 62% occurring during spike landings (8). Female athletes showed ACL injury rates 2–4 times higher than males; high school prevalence (9.3%) exceeded collegiate rates (4.4%), and outside hitters were disproportionately represented. Generic multi-sport warm-up protocols do not replicate the asymmetric single-leg spike landing central to volleyball ACL injury, making direct protocol transfer insufficient (8) (VS) (moderate evidence).

PT is the primary overuse pathology at the knee in volleyball, with a pooled prevalence of 24.8% (95% CI: 17.8–31.8%) (28), reaching 40%–50% at elite level (29). In adolescents, PT prevalence (5.8%) substantially exceeds Achilles tendinopathy (1.8%) (28). An 11-year cohort found youth-onset PT caused persistent knee functional deficits, with one in five players retiring from competitive volleyball (9) (limited evidence, single cohort).

Youth volleyball injury is thus characterized by two mechanistically linked conditions, ACL injury from acute asymmetric loading under time-critical neuromuscular conditions, and PT from chronic cumulative overload during non-uniform adaptation, both clustering in female athletes, older adolescents, and high-exposure positions, with direct implications for NMT targeting.

### 3.2 Biomechanical and neuromuscular risk factors

Four risk factor domains are particularly relevant to the adolescent volleyball context: dynamic knee valgus (DKV) and tibial internal rotation, neuromuscular fatigue, hamstring-to-quadriceps (H:Q) strength ratios, and landing strategy as a determinant of patellar tendon load.

DKV defined as the combined pattern of femoral adduction, femoral internal rotation, knee abduction, and tibial external rotation, is the most widely studied biomechanical marker within the multifactorial risk profile for non-contact ACL injury (12). It is understood as a clinically observable indicator of deficient neuromuscular control, not as an independent injury predictor. Its predictive validity as a sole screening variable is limited by substantial overlap between valgus values in athletes who do and do not sustain injury (12).

The most frequently cited prospective evidence for this mechanism comes from Hewett et al. (30). That study followed 205 female athletes across soccer, basketball, and volleyball. It was a mixed-sport sample. Volleyball players were not analysed separately. Athletes who subsequently sustained ACL rupture showed 8° greater knee abduction at landing. They also showed significantly elevated knee abduction moments compared with uninjured peers at baseline. This study provides the strongest available prospective data linking DKV magnitude to subsequent ACL injury. However, it is not volleyball-specific, and its findings apply to the adolescent volleyball population by mechanistic extrapolation rather than direct empirical confirmation. Tibial internal rotation further amplifies ACL loading. The combination of excessive knee abduction and tibial internal rotation produces the highest rates of ACL shear force during landing and cutting (12).

In volleyball, this risk pattern is expected to emerge most clearly during single-leg spike landings and unanticipated lateral block jumps. Epidemiological data confirm that 62% of volleyball ACL injuries occur during spike landings (8) (VS). The absence of anticipation in unanticipated block jumps eliminates preparatory neuromuscular activation, increasing injury risk (5) (VS). However, direct biomechanical measurement of DKV during these specific tasks in adolescent volleyball players has not been conducted. This inference is based on epidemiological data and landing mechanics evidence from adjacent sport contexts, not from dedicated DKV measurement studies in this population. Female adolescent players are specifically vulnerable. Post-pubertal females demonstrate larger knee abduction moments than pre-pubertal peers (14). Sport-specialized females also show greater post-pubertal increases in knee abduction angle (*p* = 0.005) and moment (*p* = 0.006) than multi-sport peers (31). DKV is a marker within a multifactorial risk profile, not an independent injury predictor (moderate evidence).

A systematic review of 67 studies conducted across multiple sports, not volleyball specifically found that 79% reported altered kinematics or neuromuscular outcomes following fatigue (32). Reported changes included reduced knee flexion and increased knee abduction at landing. Sex-specific differences have been identified in controlled laboratory research. Post-fatigue females tend toward greater valgus displacement. Males show compensatory increases in knee flexion (33). These patterns have not been directly replicated in adolescent volleyball populations. Additionally, standardised laboratory fatigue protocols produce inconsistent effects across biomechanical variables. They are likely to underestimate the compound effects present in real match conditions (32).

In match conditions, physical fatigue coincides with elevated cognitive load, and this interaction degrades adaptive co-activation more than either factor alone (12, 31). For volleyball players, whose spike landings follow high-intensity rallies and demand concurrent tactical decision-making, this compound effect is particularly consequential. In this sport context, fatigue functions as a risk amplifier, one that reduces neuromuscular control fidelity across a range of kinematic variables rather than producing any single, predictable change (limited evidence).

The H:Q strength ratio is widely used as a clinical screening index (34). The functional ratio (Hecc/Qcon) is more physiologically meaningful than the traditional concentric ratio (Hcon/Qcon; normative 0.6), reflecting eccentric hamstring demand during dynamic movement (35). A systematic review of 23 prospective studies found no significant association between any H:Q variant and ACL injury risk (36). For hamstring injury, associations were inconsistent. Threshold values varied widely. Substantial overlap existed between injured and uninjured athletes. These findings have not been examined specifically in adolescent volleyball players. Screening thresholds derived from adult multi-sport cohorts cannot be assumed to transfer directly to this population. Pre-season H:Q screening therefore has limited standalone predictive value in the current evidence base. Longitudinal within-athlete tracking may retain clinical utility if interpreted within a maturity-aware context. H:Q data should inform rather than determine training decisions, with this caveat explicitly acknowledged (limited evidence; adult multi-sport populations).

Landing strategy is an equally important determinant of patellar tendon load. Bisseling et al. compared symptomatic (RJK), previously symptomatic (PJK), and healthy control (CON) volleyball players during drop jumps using inverse dynamics analysis (37). PJK players showed higher knee angular velocity and ankle plantarflexion loading rates than controls (both *p* < 0.01). These differences occurred despite similar joint ranges of motion. RJK players showed a protective avoidance pattern with lower peak knee moment, power, and work than controls. This dissociation identifies a stiffer landing strategy, as a potential mechanical antecedent to PT development (37). Knee flexion angle at initial contact is inversely associated with peak vGRF and loading rate. An asymptomatic player landing with reduced knee flexion may accumulate microtrauma that manifests as symptomatic PT weeks or months later (moderate evidence) (37).

These four domains interact: fatigue worsens DKV; quadriceps dominance contributes to both ACL loading and patellar tendon strain. The biomechanical risk profile is best understood as an integrated system of interacting deficits with direct consequences for NMT design and targeting.

### 3.3 Adolescent-Specific risk: growth, maturation and the neuromuscular deficit

The risk factors identified above do not operate equally across developmental stages. During PHV, three concurrent processes create a qualitatively distinct risk profile: muscle-tendon structural mismatch, limitations of chronological age as a stratification tool, and sex-specific deterioration of neuromuscular control.

**Figure 3:**
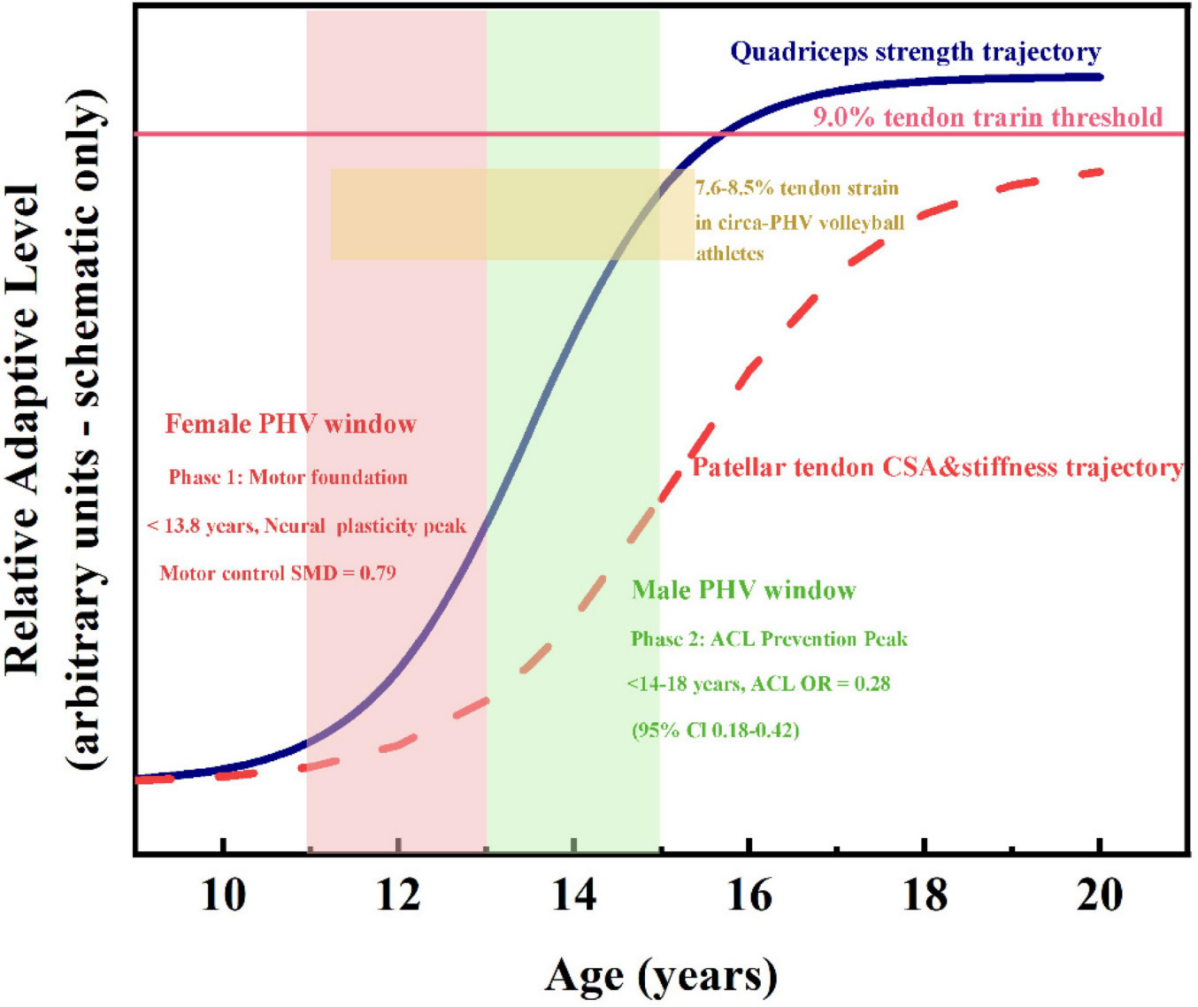
Schematic representation of the temporal mismatch between quadriceps strength and patellar tendon structural adaptation during adolescent development, with evidence based NMT intervention windows.

During PHV, bone elongates faster than the surrounding muscle-tendon unit can adapt (10, 11). Muscle mass and quadriceps strength increase substantially, but patellar tendon cross-sectional area and stiffness lag behind (38). Mersmann et al. documented this mismatch directly in adolescent volleyball athletes. Mid-adolescent players showed quadriceps strength greatly exceeding untrained peers, while patellar tendon CSA remained significantly smaller than in adult elite players with equivalent training histories. Tendon strain values during maximal contractions reached 7.6%–8.5%, approaching the approximately 9.0% threshold above which microstructural impairment becomes likely. Plyometric loading compounds this problem by driving muscle hypertrophy without reliably increasing tendon stiffness (38) (VS). The circa-PHV volleyball player therefore accumulates a chronically elevated tendon strain that neither growth nor training alone would produce (moderate evidence) (Figure 3).

PHV onset varies by ±1–2 years around population means (11, 12), meaning a single competitive age group may simultaneously contain pre-, circa-, and post-PHV athletes with qualitatively different vulnerability profiles. A scoping review of 30 studies confirmed injury incidence increases with maturity status, with growth-related injuries peaking at the growth spurt (12). Field-applicable methods, maturity offset, percentage of predicted adult height, and the Khamis-Roche equation, provide non-invasive risk stratification (11). Age-group programming systematically leaves the circa-PHV athlete unprotected at the moment of greatest tendon vulnerability (limited evidence, predominantly soccer-based cohorts).

The most consequential aspect is sex-specific divergence in neuromuscular control. Males gain coordinated strength, power, and hamstring co-activation through maturation (14). Females show the opposite: Hewett et al. demonstrated that sex differences in knee abduction moments coincide temporally with the pubertal growth spurt and are absent before it, with post-pubertal females showing decreased neuromuscular control relative to their pre-pubertal baseline (14). Reduced knee flexion at initial contact, greater frontal-plane valgus displacement, and quadriceps-dominant activation emerge after the growth spurt and coincide with peak ACL injury prevalence (15). Sport specialization accelerates this process, with specialized females showing significantly greater post-pubertal increases in knee abduction angle (*p* = 0.005) and moment (*p* = 0.006) than multi-sport peers (31) (moderate evidence).

This same window represents the optimal period for NMT. A meta-analysis found NMT risk reduction was greatest in athletes aged approximately 12–14 years, with attenuated and less consistent responses in older post-pubertal athletes (15). Movement patterns are most plastic when first being established; NMT introduced at or before circa-PHV can redirect the neuromuscular developmental trajectory, compensating for the protective maturation gains that females do not acquire automatically. Delay requires greater training volume for equivalent outcomes and forgoes the period of greatest neural plasticity (moderate evidence).

Three concurrent processes converge on the adolescent female volleyball player during the circa-PHV period: structural muscle-tendon mismatch under plyometric loading (38, 39); maturation-indexed vulnerability that chronological age cannot capture (12); and sex-specific failure to develop protective neuromuscular control (14, 15). Sport specialization and high PJT volume, without neuromuscular correction, amplify all three simultaneously and define the biological rationale for the interventions reviewed in the following section (38).

## 4 Components of effective NMT programs

### 4.1 Multimodal design principle

The decomposition of NMT into four component domains below is an analytic structure, not a prescription. No component can be meaningfully deployed alone: converging evidence establishes that multimodal NMT produces superior outcomes over any single-component intervention (40).

**Figure 4:**
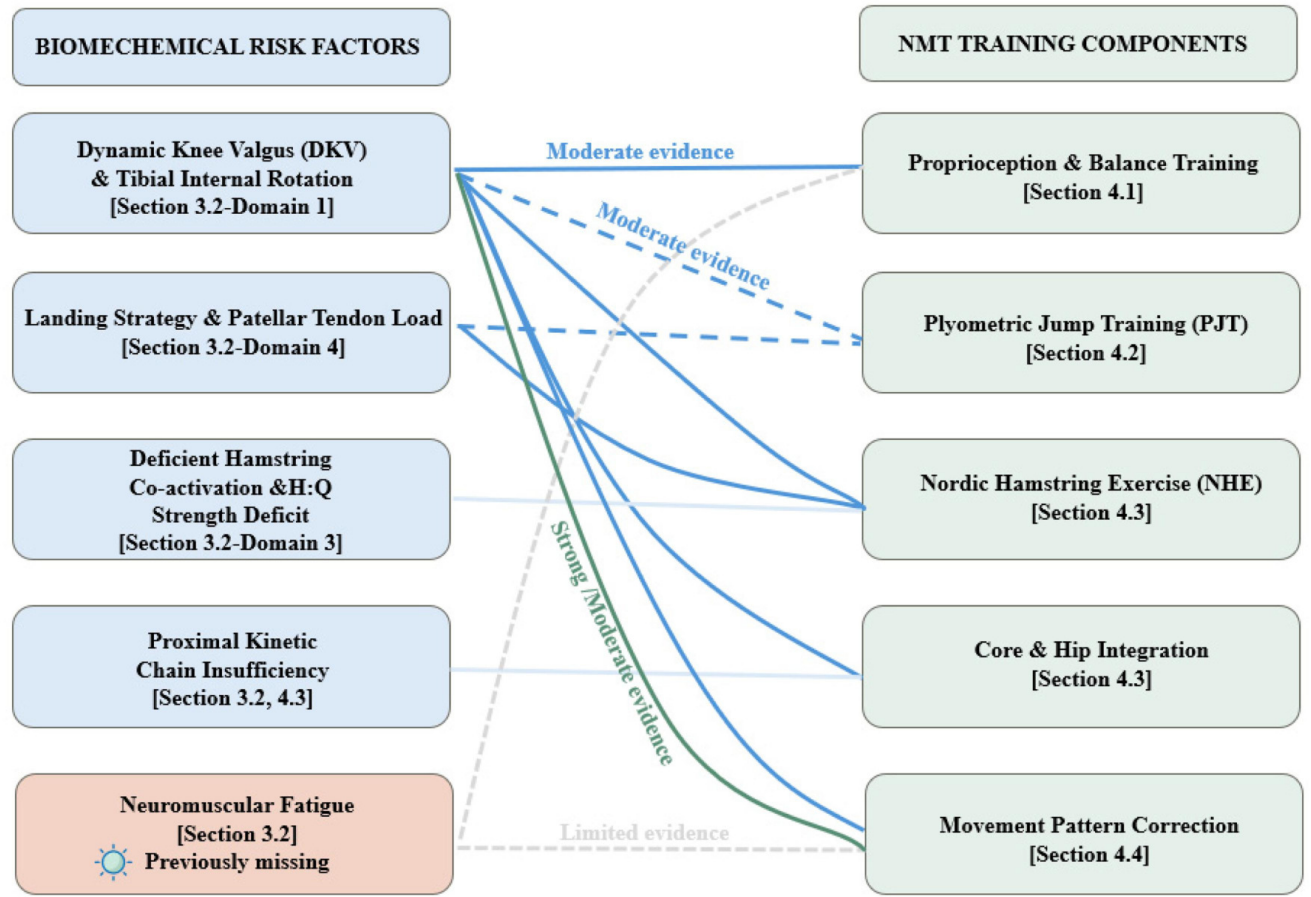
Mechanistic linkages between NMT training components and modifiable biomechanical risk factors for knee injury in adolescent volleyball players.

A meta-analysis of youth sport RCTs found multimodal injury prevention programs reduced overall injury rate by 40% with simultaneous improvements in balance, strength, and power (40, 41). A meta-regression found each additional training component per session independently reduced ACL injury risk by 17% in female athletes (42). For adolescent volleyball players, whose risk profile simultaneously includes deficits in joint position sense, tendon strain tolerance, hamstring co-activation, and landing mechanics, no single component addresses more than one dimension. Individual component analysis in subsequent sections serves mechanistic understanding; the clinical unit of deployment remains the integrated multimodal program (strong evidence) (Figure 4).

### 4.2 Proprioception and balance training

Proprioception integrates afferent signals from muscle spindles, Golgi tendon organs, and joint capsule mechanoreceptors to encode joint position, velocity, and applied force. Its functional significance in knee injury risk lies not in static joint position sense but in the speed of neuromuscular responses to perturbation. The initial 40–50 ms after ground contact precede voluntary motor activation. Joint protection in this window depends on pre-programmed responses shaped by prior sensorimotor training (12, 31). Without adequate proprioceptive input, the knee relies on passive restraints against forces the neuromuscular system cannot counter in time. For the circa-PHV female athlete, this window is further compressed by rapid limb elongation. Limb elongation transiently alters mechanoreceptor geometry before afferent accuracy can recalibrate (11).

Training on unstable surfaces shortens the neuromuscular feedback loop through peripheral and central adaptation. Repeated mechanoreceptor recruitment under perturbation increases receptor sensitivity and joint position afference signal-to-noise ratio. A systematic review of proprioceptive training learning dynamics found consistently larger and more durable gains in active joint position reproduction than non-targeted exercise, with specificity most pronounced for active, task-relevant conditions recruiting fusimotor drive and muscle spindle feedback simultaneously. Static bilateral balance tasks improve proprioceptive acuity but do not transfer fully to the single-leg, high-velocity perturbations of spike landing; progression from stable bilateral to unstable unilateral to sport-specific reactive tasks is the training architecture required for ecologically valid transfer (43).

Evidence suggests that proprioceptive training benefits may persist across full competitive seasons. Verhagen et al. (44) demonstrated this durability in volleyball players specifically (VS). However, that study targeted ankle sprain prevention rather than knee injury outcomes. Its findings therefore cannot be directly applied to ACL or patellar tendon protection at the knee. The broader durability mechanism is supported by multi-sport proprioceptive training research (43). This mechanism has not been directly examined in adolescent volleyball players at the knee joint (moderate evidence; ankle-to-knee extrapolation within volleyball; mechanistic basis from multi-sport data).

In volleyball specifically, Yang et al. reported significant improvements in dynamic postural stability following combined NMT incorporating balance elements (20) (VS). That meta-analysis received a Critically Low AMSTAR-2 confidence rating (Table 1). This finding is directional. It should be interpreted with caution. Direct volleyball-specific evidence supports these mechanisms. Zarei et al. applied the Verhagen volleyball-specific NEMEX proprioceptive balance board programme to 30 youth volleyball players (mean age 16.6 years) in a quasi-RCT design. Compared with controls, the intervention group demonstrated significant improvements in Functional Movement Screening scores, Y-Balance Test composite scores (lower quarter), and single-leg hopping performance (45). This represents the only published study implementing a volleyball-designed proprioceptive protocol in a youth population and provides grounded support for the mechanism described above (45) (VS). A systematic review of proprioceptive training effects on sport performance found concurrent improvements in explosive strength, agility, and balance in youth female volleyball players (43). The circa-PHV period is when proprioceptive recalibration is most needed. It also corresponds to the period of greatest neural plasticity. Structured proprioceptive training during this window therefore yields both corrected joint position sense and enhanced neuromuscular adaptation capacity, benefiting all other NMT components. Most mechanistic evidence for proprioceptive training derives from multi-sport populations. Direct evidence in adolescent volleyball players is limited to Yang et al. (20). That meta-analysis addresses stability outcomes broadly. It does not isolate volleyball-specific proprioceptive mechanisms. Dose parameters are currently extrapolated from football and basketball protocols. These parameters are mechanistically plausible for volleyball. The two sports share demands on joint position sense and perturbation response. However, these parameters have not been tested in adolescent volleyball players. Practical recommendations in this domain are evidence-informed. They are not evidence-confirmed for this specific sport and age group (limited to moderate evidence; Tier 2 extrapolation for dose parameters).

**Table 1:** Summary of methodological quality of tier 1 studies.

Study	Design	Quality tool	Key strengths	Key limitations	Final rating
Nunes et al. (23)	Cluster-RCT	PEDro	Random allocation; complete follow-up; ITT analysis	No blinding of participants, therapists, or assessors	6/10 — Good
Gavala et al. (24)	Controlled trial	PEDro	Baseline comparability; complete follow-up; ITT analysis	No randomization; no blinding	5/10 — Fair
Yang et al. (20)	Systematic review & meta-analysis	AMSTAR-2	PICO specified; comprehensive six-database search; appropriate statistical methods	Multiple critical domains unconfirmed; publication bias not assessed	Critically Low

Full item-level ratings for PEDro and AMSTAR-2 are provided in Supplementary Tables 1, 2 of the Supporting Information. PEDro quality grades: Excellent, 9–10; Good, 6–8; Fair, 4–5; Poor ≤ 3 (27). AMSTAR-2 confidence ratings: High, Moderate, Low, Critically Low (28). Quality ratings were not used as exclusion criteria. All three Tier 1 studies are retained in the evidence synthesis regardless of quality grade.

The proprioceptive foundation established through balance training creates the sensorimotor substrate on which plyometric loading can safely operate, the focus of the following subsection.

### 4.3 Plyometric training and landing mechanics

Plyometric training (PJT) exploits the stretch-shortening cycle (SSC) through elastic energy storage during eccentric loading and neural preactivation that elevates muscle stiffness at ground contact (46). The SSC occurs in two forms: slow (contact time >250 ms; countermovement jumps, spike approach) and fast (contact time <250 ms; block rebounds, reactive landings). Both forms are present in volleyball, justifying the inclusion of deep-countermovement and reactive drop-jump tasks in a comprehensive program. Reactive strength index (jump height/contact time) provides a sensitive monitoring tool for fast SSC adaptation (46).

Well-constructed PJT directly improves the landing biomechanics most implicated in ACL and patellar tendon injury. A meta-analysis of 13 controlled studies confirmed small-to-moderate improvements in spike jump and CMJ performance alongside improved neuromuscular coordination (46). A separate IPP biomechanics meta-analysis found significant improvements in peak knee flexion angle, reduced vGRF, and reduced knee abduction moment. Effect sizes were greatest in programs combining PJT and balance elements (47).

In volleyball specifically, Yang et al. reported significant improvements in dynamic postural stability following combined NMT incorporating balance elements (20) (VS). That meta-analysis received a Critically Low AMSTAR-2 confidence rating (Table 1). This finding is directional. It should be interpreted with caution. The mechanism is direct. PJT increases quadriceps eccentric force capacity and prolongs the deceleration phase, producing the kinematic changes that reduce ACL and patellar tendon strain (47). Volleyball-specific evidence corroborates this finding. Leporace et al. applied a six-week preventive training programme combining PJT, balance, and core stability exercises to 15 young male volleyball players (mean age 13 years, three sessions per week). Post-intervention, participants demonstrated improved sagittal plane kinematics during single-leg landing tasks and directional increases in vertical jump height, confirming that multimodal preventive programmes simultaneously improve landing mechanics and performance in an adolescent volleyball context (48) (VS). Training type specificity matters. A controlled study in post-PHV female volleyball players found that a horizontal PJT protocol failed to produce significant improvements across multiple jump and hop measures, including reactive strength index (49) (VS). Training modality within plyometrics also matters specifically for volleyball. Ruffieux et al. randomised 26 female volleyball players (aged 15–32 years) to either countermovement jump (CMJ) or drop jump (DJ) training over six weeks (two sessions per week, 60 jumps per session). CMJ training produced significantly superior gains across all jump types (17% vs. 7% on average; *p* < 0.001), demonstrating that slow stretch-shortening cycle training aligned with the spike approach is more effective for volleyball-specific jump performance than reactive fast SSC protocols (50) (VS). For youth volleyball players, this supports the primacy of CMJ-based PJT progressions before introducing reactive drop-jump variants. This suggests that PJT modality must align with the vertical and reactive demands of volleyball, rather than defaulting to horizontal or general-purpose protocols (moderate evidence).

Approximately 62% of volleyball ACL injuries occur during spike landings (8). These landings are predominantly unilateral and asymmetric, with unpredictable approach angles. Unanticipated block jumps further increase peak vGRF and elevate knee valgus moment, disrupting preprogrammed neuromuscular activation (5) (VS). Athletes trained exclusively through bilateral, anticipated, vertical PJT acquire SSC improvements that do not transfer to the asymmetric, unanticipated conditions where real-world ACL injuries occur. Combined with the circa-PHV muscle-tendon imbalance (38), a bilateral vertical-dominated program exposes the patellar tendon to increasing strain without corresponding structural adaptation. The solution is a progressive loading architecture. This begins with bilateral symmetric vertical tasks and advances through unilateral hops, lateral and diagonal bounding, reactive block-and-land sequences, and ultimately unanticipated multidirectional tasks under time pressure (5, 20) (moderate evidence).

Pooled evidence shows a larger vertical jump effect size for female than male volleyball players (ES = 1.3 vs. 0.5) (51). For circa-PHV athletes already accumulating high tendon strain from growth-related hypertrophy and sport-specific jump volume (38), PJT dose should be conservative at program onset. Landing quality supervision should take priority over volume. PJT should also be paired from the outset with the tendon-loading protocols described in Section 4.4. PJT without concurrent tendon loading risks worsening the muscle-tendon imbalance in this subgroup rather than correcting it. The biomechanical landing outcomes above derive predominantly from multi-sport adult populations (47). Direct Tier 1 replication in adolescent volleyball players has not been established. PJT volume without concurrent proprioceptive training increases demands on passive tissue restraints that an inadequately calibrated neuromuscular system cannot counter in time (moderate evidence).

Further volleyball-specific evidence comes from Dell'Antonio et al., who implemented a six-week aquatic plyometric protocol (drop jumps, two sessions per week) in 12 female youth volleyball players (mean age 16.6 years) during the pre-season period. Post-intervention, spike height improved with a large effect (ES = 1.09), squat jump with a moderate effect (ES = 0.76), and CMJ with arm swing with a moderate effect (ES = 0.78); all gains were maintained at a four-week follow-up (52) (VS). This directly mirrors the detraining-resistant adaptation reported by Nunes et al. (23) and Gavala et al. (24) and confirms the durability of plyometric-induced jump gains in adolescent female volleyball players.

While plyometric training addresses force generation and landing mechanics, the musculotendinous imbalance and proximal kinetic chain deficits that characterize circa-PHV athletes require the eccentric and core-focused interventions described in Section 4.4.

### 4.4 Eccentric resistance training and core integration

Eccentric resistance training and core stability training occupy distinct but mutually reinforcing roles within the NMT framework. Eccentric loading addresses the hamstring force deficit that leaves the ACL mechanically exposed during landing deceleration. Core stability training addresses the proximal cascade through which deficient trunk and pelvic control amplify knee loading, independent of distal neuromuscular function.

The NHE is the most extensively studied eccentric hamstring intervention. During execution, the BFlh undergoes active eccentric lengthening from approximately 85% of peak force through maximum knee extension. This produces two key adaptations: increased peak eccentric knee flexor torque (53), and BFlh fascicle elongation over 4–10-week training blocks (54). Fascicle lengthening shifts the torque-angle curve rightward, increasing force at the longer muscle lengths present at spike landing initial contact (54). This directly targets the mechanical vulnerability window in which ACL loading peaks (53).

The Hecc/Qcon ratio retains physiological meaning at the landing-phase joint angles where injury occurs (35). NHE training increases eccentric torque at precisely these critical angles (53, 54). Across 19 prospective studies (*n* = 8,459 athletes), NHE inclusion in injury prevention programs produced an overall injury risk ratio of 0.49 (95% CI: 0.32–0.74, *p* = 0.0008), reducing hamstring injuries by up to 51% (53) (moderate evidence).

#### 4.4.1 Core stability: proximal kinetic chain control

Core stability functions as a mechanistically necessary NMT component, not a supplementary one (55, 56). Ground reaction force at foot strike is transmitted proximally through the ankle, knee, and hip. Insufficient stiffness at any point in this kinetic chain shifts the force vector laterally, lengthening the lever arm relative to the knee joint centre and amplifying the knee abduction moment (55). A stronger trunk therefore provides the stable proximal platform from which hip abductors and external rotators can resist the hip adduction and internal rotation that drive the DKV pattern.

This proximal-to-distal mechanism is supported by converging evidence. Zazulak et al. (55) conducted a prospective study of 277 collegiate athletes across mixed sports. This was not an adolescent volleyball sample. Trunk displacement in response to a sudden force perturbation was significantly greater in those who subsequently sustained ACL injury. Coronal-plane lateral trunk displacement was the strongest predictor of knee ligament injury in females. This association was absent in males. This finding has not been replicated in a volleyball-specific cohort. It provides mechanistic grounds for targeting trunk neuromuscular control in female players. It does not constitute direct evidence for the adolescent volleyball population. A targeted eight-week core training RCT in jumping-and-cutting athletes produced significantly reduced knee valgus angle (*p* < 0.05), increased knee flexion, and improved trunk endurance (56). Volleyball-specific confirmation comes from Tsai et al., who applied a six-week trunk and hip programme to 16 adolescent male volleyball players (mean age 13.4 years), reporting significant reductions in trunk flexion during box landing (Cohen's d = 0.78) and in maximum knee internal rotation during spike jump landing (Cohen's d = 0.56), alongside substantial gains in isokinetic hip flexor and knee extensor strength (57) (VS). These findings collectively provide direct support for the proximal kinetic chain mechanism as a primary target in core training for adolescent volleyball players (moderate evidence).

Trunk and hip targeted NMT closes the proximal-to-distal causal loop. Myer et al. proposed trunk and hip focused NMT as a primary female athlete injury prevention component, with pilot data in uninjured high school volleyball players demonstrating meaningful hip abduction strength improvements predicted to directly reduce knee abduction loading during landing (13). For the circa-PHV female volleyball player, reduced hamstring co-activation, insufficient trunk stiffness, and underdeveloped hip abductor capacity all contribute independently to the DKV risk pattern. A program integrating NHE, core stability progressions, and hip abductor strengthening within a single session targets all three simultaneously, reflecting the physiological reality that these are biomechanically interdependent contributors to the same failure mode (moderate evidence).

As described above, circa-PHV volleyball athletes accumulate elevated patellar tendon strain through a combination of growth-related muscular hypertrophy and high PJT volume. The missing intervention component is tendon-targeted mechanical loading at the appropriate intensity and velocity to stimulate collagen turnover and stiffness adaptation without driving further strain accumulation. Heavy slow resistance training at loads producing 4.5%–6.5% tendon strain across 5 sets of 4 repetitions with a 3-second loading-unloading duration provides the mechanical stimulus for tendon structural adaptation that plyometric loading alone cannot reliably supply (38). Body-centering approaches to core stability training have also been evaluated in adolescent female volleyball populations. Kovačević et al. compared a body-centering core stability protocol with standard core exercises in female volleyball players, demonstrating that the body-centering group achieved significantly greater improvements in Berg Balance Scale scores, trunk control (*p* < 0.05), and drop jump power (*p* < 0.01), with adaptations maintained at 12-week follow-up (58) (VS). These volleyball-specific findings reinforce the functional superiority of multi-planar, perturbation-based core training over isolated strength exercises alone in this population.

These protocols, drawn from the Mersmann et al. muscle-tendon assessment and loading framework, should be incorporated into the S&C program as a complement to eccentric hamstring and core work rather than as a standalone tendon-protection module. When paired with appropriately dosed NHE and core work, tendon-targeted loading completes the strength-and-structure tier of NMT: NHE addresses distal hamstring deceleration capacity; core training addresses proximal kinetic chain stiffness; and tendon loading addresses the structural mismatch that the circa-PHV window creates. Eccentric hamstring strength and core stiffness are functionally interdependent, hamstring co-activation provides anterior tibial restraint most effectively when proximal kinetic chain stiffness prevents the lateral trunk displacement that amplifies knee abduction moment (55) (moderate evidence).

Even when muscle strength, tendon integrity, and core stability are adequate, injury can occur if the central nervous system fails to orchestrate these capacities into a correctly timed and fatigue-resistant motor programme, the coordination layer addressed in Section 4.5.

### 4.5 Neuromuscular activation and movement pattern correction

Even when joint position sense is intact, eccentric hamstring strength is adequate, and core stiffness is sufficient, injury can occur if the CNS fails to orchestrate these capacities into a correct, timely, and fatigue-resistant motor program. Neuromuscular activation and movement pattern correction training targets this coordination layer directly.

Landing from a spike or block requires co-activation across functionally antagonistic muscle groups within a time window beginning before foot contact and largely resolved within 40–50 ms of ground interaction (11, 31). This pre-activation window is governed by feed-forward programming, not reactive feedback (12, 31). NMT targets this system by repeatedly exposing athletes to controlled perturbations requiring anticipatory joint stiffness and co-activation, progressively reducing cognitive load and driving correct multi-joint activation toward automaticity (40, 59). For post-pubertal female athletes, who have not acquired the protective co-activation patterns that males develop through maturation (14), structured NMT provides the training stimulus substituting for the developmental neuromuscular spurt that does not spontaneously occur (14, 15). Evidence from NMT intervention studies confirms meaningful reductions in dynamic knee valgus angles, optimized vastus medialis obliquus/vastus lateralis synchrony, and decreased anterior tibial shear forces through improved post-contact muscle latency (59), neural timing adaptations indicating the motor program has been restructured centrally, not merely that muscles have been strengthened (moderate evidence).

This cueing effect has been directly demonstrated in female volleyball players. Slovák et al. recruited 10 female volleyball players (mean age 20.4 years) and compared drop landings under external focus (land as softly as possible), internal focus (flex your knees on landing), and no-instruction conditions using statistical parametric mapping of the full landing time series. External focus instructions produced significantly reduced vertical ground reaction force and lower sagittal flexion moment during the first 30% of the landing phase, while no instruction produced no biomechanical change (60) (VS). This is the only published study replicating this cueing effect in a volleyball-specific landing task and directly validates the practical instruction recommendations in Section 6.

Fatigue degrades motor program stability in ways that current NMT designs do not consistently address (32). As established in Section 3.2, the fatigue-unanticipated movement interaction is most hazardous under match conditions where concurrent cognitive load is elevated. Laboratory fatigue protocols underestimate this compound effect (32). Delivering NMT landing components periodically under controlled fatigue conditions directly targets this gap (limited evidence, predominantly from non-volleyball populations). Movement pattern correction using video and verbal feedback has been validated in adolescent female volleyball athletes specifically. Parsons and Alexander provided feedback sessions to 19 female volleyball players aged 12–14 years and assessed landing kinematics at baseline, immediately post-feedback, and at two and four weeks. The intervention group demonstrated significantly increased ankle dorsiflexion, knee flexion, hip flexion, and trunk flexion angles compared with controls at the four-week assessment (*p* ≤ 0.05), confirming that technique feedback produces durable kinematic changes in spike landing at precisely the developmental age identified in Section 3.3 as the period of greatest motor plasticity (61) (VS).

The role of cognitive cueing strategies in movement pattern correction merits specific discussion, though the evidence base in volleyball-specific adolescent populations remains limited. A Bayesian network meta-analysis synthesizing data from 22 RCTs involving 878 athletes found that external focus instructions produced the largest improvements in peak knee flexion angle during jump-landing tasks (mean difference = 26°; credible interval: 7.5°–44°; SUCRA = 0.94), ranking above all other tested intervention components for this specific outcome (59). The mechanistic interpretation aligns with the constrained action hypothesis: external focus instructions direct attention toward the intended effect of movement in the environment, enabling more efficient subcortical automaticity, whereas internal focus instructions increase cortical engagement and can interfere with the fluid execution of pre-programmed motor patterns (59). For movement pattern correction in practice, instructional language choices may produce different biomechanical outcomes even when the target movement change is identical. Cueing an athlete to “land as quietly as possible” may produce greater knee flexion than “flex your knee on landing”, not because the athlete is performing a different movement but because the attentional mechanism engaged is different (59). (limited evidence).

Reactive NMT has also shown promise in volleyball players with established neuromuscular deficits. Seyedahmadi et al. randomised 30 male volleyball players with a history of ACL reconstruction to six weeks of reactive NMT (perturbation-based balance and proprioceptive tasks, three sessions per week) or controls. The RNMT group demonstrated significant improvements in dynamic balance and functional performance relative to controls (62) (VS). Although this population is post-reconstruction rather than healthy adolescents, the findings demonstrate that volleyball-specific reactive NMT corrects the movement-pattern deficits most relevant to the injury scenarios described in this section.

## 5 Synergy of injury prevention and athletic performance enhancement

### 5.1 Effects on vertical jump height and agility

NMT simultaneously serves injury prevention and performance objectives. The proprioceptive recalibration, multi-joint co-activation, eccentric hamstring strength, and core stiffness developed through NMT determine force absorption and redirection per ground contact cycle, the injury-protective and performance-enhancing effects are the same physiological outputs expressed in different task contexts. Ramirez-Campillo et al. synthesized 14 controlled volleyball studies, reporting a moderate pooled effect for vertical jump height (Cohen's d = 0.82; I^2^ = 34.3%), with a larger female-specific estimate (ES = 1.3 vs. 0.5 in males) (51) (VS). A subsequent meta-analysis confirmed small-to-moderate improvements in spike jump and CMJ, though fewer than three included studies assessed agility or change-of-direction (46) (VS). Neither meta-analysis isolated the incremental contribution of injury-prevention NMT components over PJT alone, a critical distinction given that PJT alone does not constitute the full NMT model. Two Tier 1 studies provide direct evidence in adolescent volleyball populations. Nunes et al. conducted a 20-week cluster-randomized controlled trial in 32 novice youth players with a mean age of 13 years (23). This study received a PEDro score of 6/10, rated Good (Table 1). Integrative NMT produced significant CMJ improvements at 6 weeks (*p* < 0.005). Further gains were observed at 12 weeks (*p* < 0.001). Improvements were maintained through an 8-week detraining period. This indicates structural durability of the adaptation (23) (VS).

Together, these two studies provide the primary Tier 1 evidence base for performance outcomes. One was rated Good and one was rated Fair on PEDro. The intervention group showed significantly greater improvements than controls in CMJ, medicine ball throw, and change-of-direction time (group interaction *p* < 0.001 for all outcomes). Change-of-direction gains were maintained through an 8-week detraining period (24).

Both populations correspond to the developmental window identified in Section 3 as the period of greatest NMT responsiveness.

Wan et al. (63) synthesised 17 RCTs involving 649 young athletes across multiple sports. Volleyball was not the primary sport studied. Integrative NMT produced significantly greater improvements than traditional physical fitness training across three outcomes. Jumping performance improved (SMD = 0.53, 95% CI: 0.32–0.73, I^2^ = 0.0%). Sprinting capacity improved (SMD = −0.76, 95% CI: −1.13 to −0.39, I^2^ = 57.6%). Maximal strength also improved (SMD = 1.01, 95% CI: 0.35–1.67, I^2^ = 81.9%).

Heterogeneity differed substantially across these three outcomes and requires separate consideration. Jumping performance showed no heterogeneity (I^2^ = 0.0%). The pooled SMD of 0.53 is the most reliable of the three estimates.

Sprint capacity showed substantial heterogeneity (I^2^ = 57.6%; Section 2.3 threshold: 50%–75% = substantial). The 17 RCTs spanned multiple sports. Each sport imposes different movement and sprint demands. NMT programme designs also differed across trials. These factors likely contributed to between-study variance. One important observation can be made from the data directly. The confidence interval lies entirely below zero (95% CI: −1.13 to −0.39). All studies therefore favoured NMT for sprint capacity. Direction is consistent. However, the magnitude of SMD = −0.76 should be treated with caution. It reflects a wide range of underlying study effects.

Maximal strength showed considerable heterogeneity (I^2^ = 81.9%; Section 2.3 threshold: >75% = considerable). This is the highest I^2^ value in Table 2. The confidence interval spans 1.32 SMD units (95% CI: 0.35–1.67). This width directly reflects high between-study variability. Strength measurement approaches likely differed across the 17 RCTs. No moderator analysis for this outcome was reported in Wan et al. (63). The pooled SMD of 1.01 should not be used as a benchmark. It is the least reliable estimate in Table 2. It should be treated as directional only (multi-sport Tier 2 evidence; considerable heterogeneity).

**Table 2:** Key quantitative outcomes from cited meta-analyses and controlled trials.

Outcome Domain	Source	Population	Studies (n)	Effect Size	95% CI	I^2^	Evidence Grade
Dynamic stability	Yang et al. (20)	Volleyball athletes	7 RCTs	SMD = 0.63	0.35–0.90	67.5%	Limited (AMSTAR−2 Critically Low)
Vertical jump height	Ramirez-Campillo et al. (51)	Volleyball players	14 studies	Cohen's d = 0.82	-	34.3%	Moderate
Jumping performance	Wan et al. (63)	Young athletes	17 RCTs	SMD = 0.53	0.32–0.73	0.0%	Moderate
Sprint capacity	Wan et al. (63)	Young athletes	17 RCTs	SMD = −0.76	−1.13 to −0.39	57.6%	Moderate
Maximal strength	Wan et al. (63)	Young athletes	17 RCTs	SMD = 1.01	0.35–1.67	81.9%	Moderate
Motor control	Williams et al. (65)	Youth athletes (8–18y)	9 studies	SMD = 0.79	0.38–1.20	-	Limited- Moderate
ACL injury risk (all ages)	Myer et al. (15)	Female athletes	14 trials	OR=0.54	0.35–0.83	-	Moderate
ACL injury risk (14–18y)	Myer et al. (15)	Female athletes	14 trials	OR=0.28	0.18–0.42	-	Moderate
Hamstring injury	van Dyk et al. (53)	Mixed athletes (*n* = 8,459)	19 studies	RR = 0.49	0.32–0.74	-	Moderate
Knee injury prevention	Kong et al. (69)	Mixed athletes (*n* = 28,176)	19 RCTs	RR = 0.75	0.65–0.85	-	Moderate

Whether these effect sizes apply to adolescent volleyball players requires confirmation from volleyball-specific trials. These findings provide directional support. They should not be treated as directly applicable performance benchmarks for the target population. No significant between-group difference was found for agility on standardized tests. This reflects the limited sensitivity of conventional agility tests to the reactive, visuomotor demands central to volleyball. It does not indicate an absence of neuromuscular agility benefit (moderate evidence) (63).

Key moderators of CMJ effect size are maturity status, baseline training level, program duration, and NMT integration (46, 51). Younger and less trained athletes show the largest gains; more experienced athletes show smaller and less consistent improvements (46). Program durations of 8–12 weeks appear sufficient for meaningful CMJ and change-of-direction gains that persist through subsequent detraining periods, supporting pre-season loading rather than in-season supplementary volume (23, 45) (moderate evidence).

### 5.2 Effects on dynamic stability in volleyball-specific tasks

Dynamic stability underpins every technical action in volleyball, and failure to maintain neuromuscular control during landing transitions constitutes a primary non-contact injury mechanism. The most comprehensive evidence comes from Yang et al. (20), a 2026 systematic review and meta-analysis synthesizing seven RCTs (2016–2024) across Turkish, Iranian, Serbian, and Tunisian volleyball populations.

The pooled estimate from Yang et al. was SMD = 0.63 (95% CI 0.35–0.90; I^2^ = 67.5%) (20) (VS). By Cohen's conventions, this SMD value falls within the moderate effect magnitude range. Substantial heterogeneity was present (I^2^ = 67.5%; Section 2.3 threshold: 50%–75% = substantial). This level of heterogeneity has direct consequences for interpretation. The pooled SMD of 0.63 is an average across seven studies with meaningfully different results. It is a directional indicator. It should not be used as a precise population-level effect size.

Six sources of heterogeneity can be identified from information in the manuscript. First, the seven RCTs came from four national contexts: Turkey, Iran, Serbia, and Tunisia. Training culture and baseline fitness levels differ across these settings. Second, NMT programme components were combined in different proportions across studies (Table 3). Third, participant age was not uniformly reported across included RCTs (Table 3). Fourth, studies were published over an eight-year span from 2016 to 2024. Programme design standards evolved during this period. Fifth, sex composition was mixed. The subgroup analysis in Yang et al. (20) confirmed that female athletes showed more pronounced lower-limb stability gains than males. Sex is therefore a confirmed moderator. It directly contributes to the observed I^2^ of 67.5%. Sixth, different outcome measurement tools were used across the seven studies. Variation in measurement instruments adds a further layer of between-study variance.

**Table 3:** Characteristics of tier 1 studies included in the primary evidence synthesis.

Study	Design	n	Age	Sex	Intervention	Duration	Key Outcomes Reported
Yang et al. (20)	Systematic review & meta-analysis (7 RCTs)	7 RCTs	Not uniformly reported across included RCTs	Mixed	Combined NMT incorporating balance, plyometric, and stability elements	Studies published 2016–2024	Dynamic stability: SMD = 0.63 (95% CI 0.35–0.90; I^2^ = 67.5%) Subgroup: females showed greater lower-limb stability gains than males
Nunes et al. (23)	Cluster-RCT	32	Mean 13 years (novice players)	Mixed	Integrative NMT (multimodal; balance + plyometric + strength + technique)	20 weeks	CMJ: significant at 6 weeks (*p* < 0.005) and 12 weeks (*p* < 0.001) Gains maintained after 8-week detraining period
Gavala et al. (24)	Controlled trial with comparator group	61	Pre-/early-pubescent	Female	Integrative NMT (in-season programme)	12 weeks	CMJ, medicine ball throw, COD time: group × time interaction *p* < 0.001 for all outcomes COD gains maintained after 8-week detraining period

Tier 1 classification requires adolescent volleyball players as the primary study population with reported NMT biomechanical or performance outcomes (Section 2.2). Age stratification by maturity status was not reported in any included study. Yang et al. (20) is a meta-analysis of seven RCTs; individual RCT-level characteristics are not fully extractable from the source publication. .

CMJ, countermovement jump; COD, change of direction; NMT, neuromuscular training; RCT, randomised controlled trial; SMD, standardised mean difference.

The methodological quality of this meta-analysis is Critically Low by AMSTAR-2 (Table 1). Under the grading system applied in this review, this corresponds to limited evidence. The substantial heterogeneity further limits confidence in the pooled estimate. The SMD of 0.63 should be treated as a directional indicator only. It should not be interpreted as a confirmed population-level effect size (limited evidence; AMSTAR-2 Critically Low; substantial heterogeneity).

Subgroup analyses by body region and sex revealed meaningfully different adaptation patterns between male and female athletes. Female athletes exhibited more pronounced NMT-induced gains in lower-limb stability than their male counterparts (20). This finding is mechanistically coherent with the greater baseline neuromuscular deficit that females carry into adolescence and early adulthood, as described above. Plyometric-dominant NMT components reduce dynamic knee valgus moments during landing (47), directly addressing the key biomechanical precursor to ACL injury (31). Proprioceptive loading additionally enhances plantar mechanoreceptor sensitivity, improving feedforward postural adjustment speed during single-leg contact phases (20). These lower-limb adaptations carry direct clinical significance. In male collegiate volleyball players, Y Balance Test interlimb asymmetry in the anterior reach direction was significantly correlated with non-contact injury incidence (r = 0.597, *p* < 0.01), while composite scores correlated with isokinetic lower-limb strength, jump height, and agility performance across multiple directions (64) (VS) (moderate evidence).

Although this dataset was male, the mechanistic link between corrected asymmetry and reduced injury risk is likely generalizable across sexes, and supports the value of lower-limb NMT as a primary injury-prevention tool in female volleyball players.

Male athletes demonstrated comparatively greater gains in upper-limb stability following NMT (20); mixed-sex program should account for these sex-differentiated patterns. The sex-differentiated stability response to NMT confirms that a uniform protocol delivered indiscriminately across mixed-sex teams is unlikely to be optimal for either group. Dynamic stability improvements are not merely performance-enhancing, they represent a direct reduction in the modifiable risk factors that predispose volleyball athletes to knee and ankle injury. Dosing parameters reported in Yang et al. (20) include sessions of 16–20 min. These are delivered two to three times per week over four to six months. They are integrated into the existing warm-up or conditioning block. This duration and frequency are broadly consistent with the multimodal design principles in Section 4 and the proprioceptive learning timelines reviewed above (20, 40). Yang et al. (20) received a Critically Low AMSTAR-2 rating (Table 1). These dosing figures are therefore indicative. They are not empirically established benchmarks for adolescent volleyball players (limited evidence; AMSTAR-2 Critically Low; no volleyball-specific dose-response trial available).

### 5.3 Sex-Specific and Age-specific adaptations

**Figure 5:**
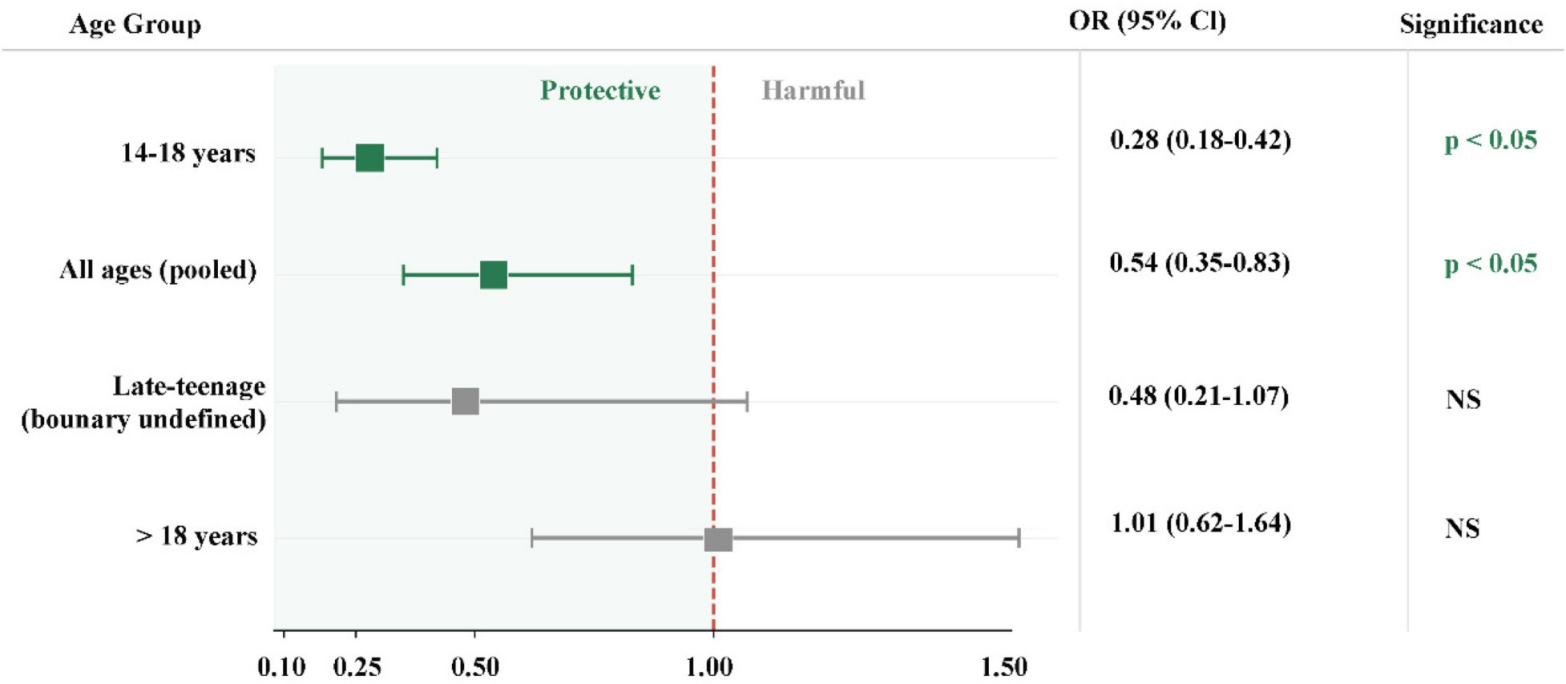
Age-stratified protective efficacy of NMT against ACL injury in female athletes.

The protective efficacy of NMT against ACL injury in female athletes is not uniformly distributed across the adolescent age range. A meta-analysis of 14 prospective controlled trials found that NMT reduced ACL injury risk by approximately half compared with controls (OR=0.54, 95% CI 0.35–0.83) (15). Stratification by age revealed a pronounced gradient. In mid-teenage female athletes, the protective effect was substantially stronger than in the pooled sample (OR=0.28, 95% CI 0.18–0.42). In late-teenage athletes, the effect was attenuated and no longer statistically significant (OR=0.48, 95% CI 0.21–1.07). In female athletes above 18 years, no meaningful risk reduction was detectable (OR=1.01, 95% CI 0.62–1.64) (15) (Figure 5). This gradient follows directly from the developmental biology described above. The mid-teenage period corresponds to the window in which maladaptive landing mechanics are consolidating but have not yet been automated through thousands of repetitions. NMT introduced before this consolidation redirects the developing motor program. NMT introduced after it must overcome an entrenched pattern at greater training cost and with less complete correction (moderate evidence) (15).

A complementary dataset addresses age and body size as moderators of NMT efficacy specifically for motor control, independent of injury incidence. A systematic review and meta-analysis of nine controlled studies in youth athletes aged 8–18 years found that bodyweight-based NMT produced a moderate, significant overall improvement in motor control tasks including dynamic balance and movement quality (SMD = 0.79, 95% CI 0.38–1.20). Moderator analyses revealed the gradient ran in the opposite direction from chronological age: program were more effective in athletes younger than 13.8 years and shorter than 162.6 cm than in older and taller peers, and effect sizes were larger in lighter than in heavier individuals (65). Younger, pre-pubertal athletes are acquiring motor patterns in a nervous system with greater structural capacity for long-term potentiation, meaning that the same NMT stimulus produces more durable neural encoding than equivalent training delivered post-pubertally (65). The pre-PHV volleyball player accordingly represents the highest-yield entry point for motor control development. Program introduced at this stage build the neuromuscular substrate on which all subsequent technical and injury-prevention training is layered (limited to moderate evidence) (65).

These two datasets define a sequential intervention logic: motor foundation work before 13.8 years where neural plasticity is greatest (65), and injury-prevention intensity maximized in the 14–18-year window where ACL risk reduction is strongest (15). A single programmed architecture can serve both phases if periodized by maturity status rather than calendar age (39) (moderate evidence).

## 6 Translational strategies for strength and conditioning coaches

### 6.1 Evidence-Based program design: dose-response considerations

These dose-response parameters derive predominantly from football-based multi-sport trials; no volleyball-specific RCT has examined dose-response relationships for NMT in adolescent players. Meta-analytic evidence supports dosing recommendations across four dimensions: session duration, weekly frequency, programmed length, and total training volume, each independently influencing whether protective effects are realized.

Session duration of 10–20 min is sufficient to reduce lower-extremity injury risk. Sessions of 10–15 min produced injury risk ratios comparable to longer sessions (IRR = 0.55, 95% CI 0.42–0.72 vs. IRR = 0.60, 95% CI 0.46–0.76) (66). Sessions exceeding 20 min produced greater ACL injury reduction in female athletes. Effective trials averaged approximately 24 min per session (67, 68). For adolescent volleyball players constrained by court scheduling, 15–20 min delivered as a structured warm-up replacement represents the most operationally feasible target (20, 40). Sessions below 10 min have not produced consistent protective effects in the multi-sport trial literature. They should not be considered adequate based on current evidence. No dose-threshold study has been conducted specifically in adolescent volleyball players (moderate evidence; multi-sport extrapolation) (66).

A weekly frequency of two to three sessions is the most robustly supported dosing parameter. Two sessions per week reduced injury incidence substantially (IRR = 0.50, 95% CI 0.29–0.86). Three sessions produced the largest pooled risk reduction (IRR = 0.40, 95% CI 0.31–0.53) (66). Multi-session program consistently outperformed single-session designs in ACL injury reduction (OR=0.35 vs. OR=0.62) (67). Embedding NMT into two to three existing sessions represents the most operationally feasible approach for volleyball squads. This approach maintains the frequency threshold identified in multi-sport trials. No frequency-comparison study has been conducted in adolescent volleyball players specifically (moderate evidence; multi-sport extrapolation).

Eight weeks is the minimum program length required to produce measurable neuromuscular adaptation (42). Longer program yields greater ACL protective effects in female athletes (long vs. short duration: OR=0.35 vs. OR=0.61) (67). Program exceeding 26 weeks produced the largest pooled effect size across 19 RCTs involving 28,176 participants (RR = 0.75, 95% CI 0.65–0.85) (69). The practical recommendation is an initial loading phase of at least eight weeks. Eight weeks is the minimum programme length associated with measurable neuromuscular adaptation in multi-sport trials. Whether this threshold applies without modification to adolescent volleyball players cannot be confirmed from current evidence (moderate evidence; Tier 2 extrapolation; no volleyball-specific dose-response data available).

**Figure 6:**
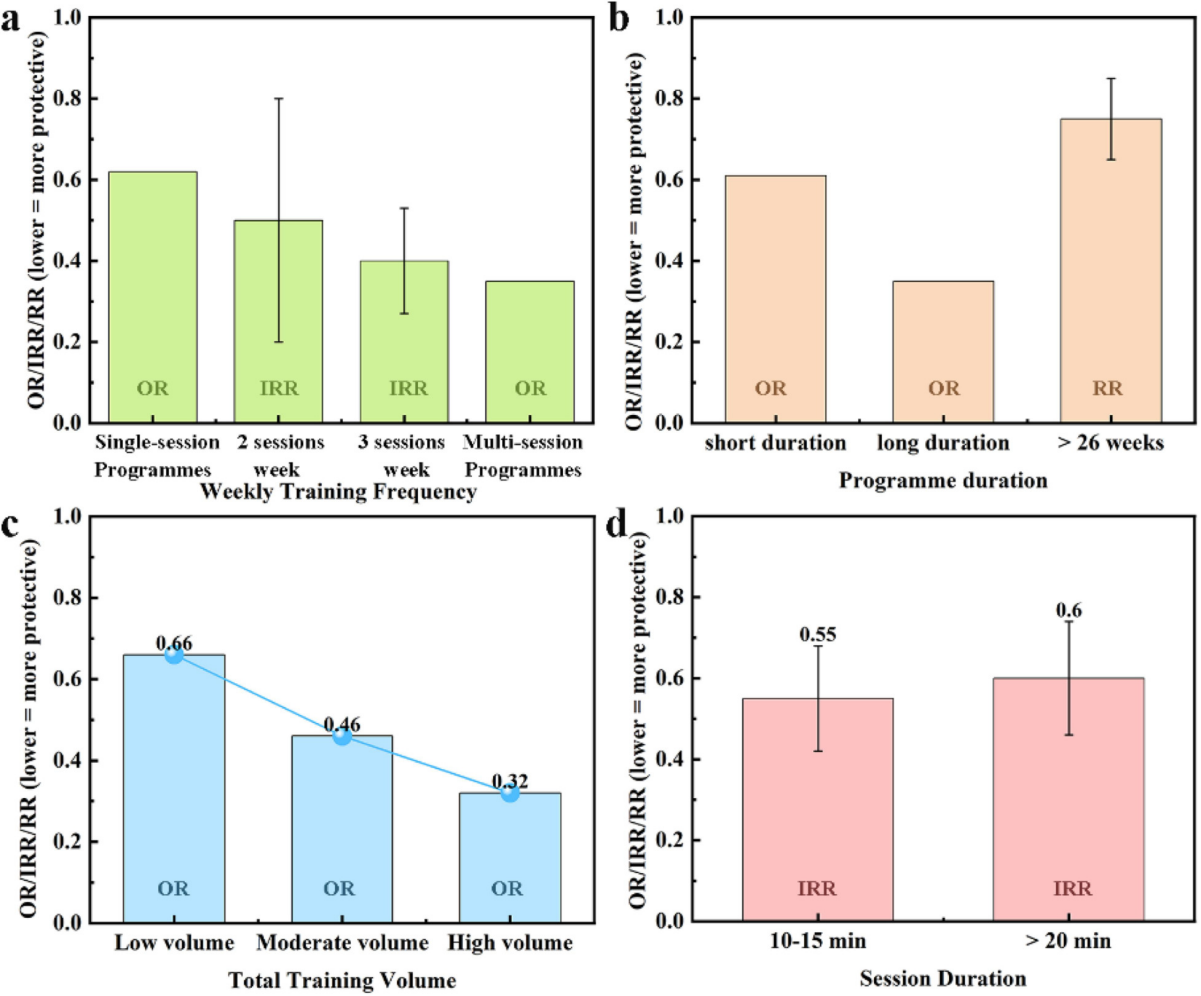
Dose-response relationships between NMT program parameters and injury risk reduction.

Total training volume shows a clear dose-response relationship: low-volume NMT reduced ACL risk modestly (OR=0.66), moderate volume strengthened the effect (OR=0.46), and high-volume program produced the largest reduction (OR=0.32) (68). Effective ACL prevention program accumulated a mean total of 18 h (68), 2–3 sessions/week, 30 + week volleyball program without displacing technical development. Maintaining frequency and continuity across a full season produces greater cumulative benefit than maximizing session duration at the cost of adherence (strong evidence from multi-sport populations; moderate evidence in the volleyball-specific context) (Figure 6).

### 6.2 Implementation fidelity and compliance

Dose parameters only produce protective effects if the program is delivered as intended, compliance is a primary determinant of outcome. This relationship is non-linear. Evidence from multi-sport female athlete trials shows that low compliance was associated with a 4.9-fold increase in ACL injury risk compared with high compliance (68). Moderate compliance was associated with a 3.1-fold increase. These figures are not drawn from volleyball-specific or adolescent-specific data. The precise magnitudes may not transfer directly to this population. The directional implication is clear and consistent with neuromuscular adaptation logic. Partial adherence provides substantially less than proportional protection. This principle is likely to apply across sport contexts. An athlete completing 60% of scheduled sessions does not receive 60% of the protective benefit.

Supervision quality independently modifies outcome: each additional NMT component per session reduced ACL injury risk by 17% in female athletes (42). Reducing session complexity to improve adherence is counterproductive, removing mechanistically necessary components undermines the protection the dose parameters are designed to deliver.

Three practical measures support compliance in youth team sport. First, integrating NMT into mandatory warm-up removes the scheduling barrier most commonly associated with dropout. Second, maintaining brief session format reduces the perceived time burden. Third, applying progressive difficulty increases across the season prevents habituation and sustains athlete engagement (68). For adolescent volleyball players specifically, framing NMT as a performance development tool rather than an injury prevention protocol increases voluntary uptake among athletes and coaching staff (moderate evidence).

### 6.3 Injury risk minimization protocols

Injury risk in adolescent volleyball players fluctuates with maturation stage, training load, and neuromuscular control fidelity under fatigue. When all three co-occur, their interaction is multiplicative rather than additive. The circa-PHV athlete accumulating elevated tendon strain, training at high plyometric volume without tendon loading, attending only a fraction of NMT sessions, and landing repeatedly under fatigue accumulates risk across all dimensions simultaneously. Program architecture must address not just each risk factor in isolation, but the conditions under which they converge.

Within a single competitive age group, athletes may span pre- to post-PHV. Each subgroup carries a qualitatively distinct vulnerability profile that chronological age cannot identify. Maturity offset, %PAH, and the Khamis-Roche equation provide non-invasive flagging and should be collected twice per season (10, 12). Once flagged, three load adjustments apply: supplementary PJT volume held at approximately 120 jumps per week across two sessions; eccentric and isometric tendon loading paired with every PJT session; and observational landing quality monitoring at each session. This monitoring is necessary because the circa-PHV tendon strain profile changes across growth episodes of weeks rather than months (moderate evidence) (12).

Compliance and supervision requirements are addressed in Section 6.2 and apply equally here. Fatigue amplifies injury risk by degrading neuromuscular control fidelity under the unanticipated movement demands of match conditions (32). As established in Section 4.5, this effect is most pronounced when physical fatigue coincides with elevated cognitive load. Two design principles follow. First, NMT landing components should periodically be delivered under controlled fatigue conditions. Second, high-volume PJT should not be scheduled at the end of long technical sessions (moderate evidence) (14, 32).

Maturity-indexed load management, compliance-maximizing program integration, and fatigue-aware session sequencing address overlapping risk dimensions that compound when they co-occur. The S&C coach's role is to reduce the intersection of these factors through deliberate program architecture.

### 6.4 Targeted conditioning for At-risk subgroups

Two subgroups within the adolescent volleyball population carry identifiably higher and mechanistically distinct injury risk profiles. Both warrant supplementary conditioning beyond the standard program. The first group is pubertal female athletes with insufficient trunk and hip neuromuscular control. The second is outside hitters, whose positional role imposes systematically higher single-leg landing exposure than any other position. These subgroups are not mutually exclusive. For athletes who belong to both, the conditioning priorities described below apply concurrently.

Pubertal female athletes are the higher-priority subgroup for trunk and hip-focused conditioning. Post-pubertal females do not acquire the progressive hip abduction strength and hamstring co-activation gains that males gain through maturation. This produces quadriceps dominance, increased hip adduction, and frontal-plane loading that converges on the knee (14). Trunk displacement amplifies this risk. Prospective data from 277 collegiate athletes showed that deficits in trunk neuromuscular control were significant predictors of ACL injury in females but not males (55). Core training targeting trunk and hip co-activation simultaneously reduces knee valgus torque, hip adduction angles, and trunk lateral displacement. This is the mechanical pathway from proximal insufficiency to distal knee overload (56). Targeted trunk and hip NMT should incorporate hip abductor strengthening, gluteal activation, single-leg squats with trunk stability constraints, lateral resistance band exercises, and single-leg Romanian deadlift progressions. These exercises address lower-limb alignment during landing and the specific post-pubertal strength deficit that male maturation provides automatically (13). This block should be structured as a prioritized component within the multimodal NMT program. It should progress from stable bilateral to unstable unilateral to dynamic perturbation tasks and begin at or before the circa-PHV window, when the deficit is first emerging (moderate evidence) (13, 14, 55, 56).

Outside hitters require a conditioning emphasis that standard bilateral landing curricula do not provide. Systematic review evidence confirms that outside hitters exhibit the highest ACL injury incidence of any positional role in volleyball. This is driven by high-velocity spike landings involving unilateral ground contact, lateral trunk displacement, and unpredictable approach angles (8). Single-leg post-spike landing accounts for the majority of contacts in match conditions. It produces a mean knee flexion angle approximately 13.8° smaller than bilateral landing, with higher vertical ground reaction forces concentrated in the 30–50 ms post-contact window (70) (VS). Non-dominant side landings generate significantly higher knee valgus moments than dominant-side contacts. This is consistent with the greater trunk lateral bending that occurs when the ball is set to the non-dominant position (70) (VS). Standard bilateral jump-and-land drills do not train the neuromuscular system in this asymmetric, high-velocity configuration. Improvements in bilateral landing mechanics do not transfer reliably to the actual injury-risk scenario.

The conditioning response is a progressive single-leg landing curriculum. It begins with hop-to-stabilize drills on the non-dominant leg and advances through lateral and diagonal single-leg bounds and spike-approach landings with external focus cues targeting knee flexion depth. The final stage incorporates unanticipated set-direction variation. The non-dominant leg should receive at least equal training volume, given its documented disproportionate valgus loading (70). No prospective RCT has evaluated a position-specific single-leg landing program in outside hitters. This recommendation is therefore graded as expert-informed practice based on biomechanical rationale, pending direct empirical validation (limited evidence) (8, 70, 71, 72).

### 6.5 Summary of key recommendations for practitioners

The following five points distil the translational evidence from Sections 6.1–6.4 into practical guidance for coaches and strength and conditioning practitioners working with adolescent volleyball players.

Programme dose. Deliver NMT as a structured warm-up of 15–20 min per session. Aim for two to three sessions per week. Maintain the programme for a minimum of eight weeks. Continuing delivery across the full competitive season produces the greatest cumulative protective benefit.

Compliance strategy. Integrate NMT into the mandatory warm-up. This removes the scheduling barrier most commonly associated with dropout. Frame the programme as a performance development tool rather than an injury prevention protocol. This framing increases voluntary uptake among athletes and coaching staff.

Maturity monitoring. Assess biological maturity status using field-applicable methods twice per season. Suitable tools include maturity offset, percentage of predicted adult height, and the Khamis–Roche equation. For circa-PHV athletes, cap supplementary plyometric volume at approximately 120 jumps per week across two sessions. Pair every plyometric session with eccentric and isometric tendon loading to address the muscle-tendon structural mismatch characteristic of this developmental stage.

Pubertal female athletes. Prioritise trunk and hip neuromuscular conditioning for pubertal female players. Include hip abductor strengthening, gluteal activation, single-leg squats with trunk stability constraints, lateral resistance band exercises, and single-leg Romanian deadlift progressions. This subgroup does not acquire protective co-activation patterns automatically through maturation. Targeted supplementary conditioning is therefore required.

Outside hitters. Outside hitters carry the highest ACL injury exposure of any positional role. Implement a progressive single-leg landing curriculum. Begin with hop-to-stabilise drills on the non-dominant leg. Advance through lateral and diagonal bounds and spike-approach landings with external focus cues targeting knee flexion depth. The final stage should incorporate unanticipated set-direction variation. Give the non-dominant leg at least equal training volume given its disproportionate valgus loading.

## 7 Conclusion and future directions

### 7.1 Summary of evidence

This review addressed two research questions. RQ1 asked whether NMT reduces the biomechanical risk factors for knee injury in adolescent volleyball players. RQ2 asked whether the same intervention simultaneously improves athletic performance in this population.

#### 7.1.1 RQ1: biomechanical risk factor reduction

The available evidence supports the conclusion that multimodal NMT reduces the primary biomechanical risk factors for knee injury in adolescent volleyball players. Across controlled studies, NMT consistently improved peak knee flexion angle at landing, reduced peak vertical ground reaction forces, and reduced knee abduction moment. Tendon-targeted loading protocols address the muscle-tendon structural imbalance that characterizes circa-PHV athletes. This is a deficit that plyometric training alone cannot resolve. These biomechanical gains are most pronounced when plyometric, balance, eccentric strength, and technique components are integrated within a single multimodal program.

Most direct evidence derives from Tier 2 sources; the only volleyball-specific NMT meta-analysis identified seven RCTs, predominantly stability-outcome studies rather than detailed biomechanical landing analyses. Tier 1 evidence in the adolescent volleyball population remains sparse. Practical recommendations should be treated as evidence-informed extrapolations rather than evidence-confirmed prescriptions (limited to moderate evidence).

Age-specific findings reinforce the importance of early intervention. NMT produces its strongest ACL injury risk reduction in female athletes aged 14–18 years. The effect is absent above age 18. Motor control improvements are greatest in athletes below 13.8 years of age. The adolescent developmental window is simultaneously the period of greatest injury risk and greatest NMT responsiveness. Delayed implementation carries a demonstrable and quantifiable cost in protective efficacy (moderate evidence).

#### 7.1.2 RQ2: athletic performance enhancement

NMT produces meaningful and durable performance improvements in adolescent volleyball players. Evidence quality differs by outcome domain. The two domains must be assessed separately.

For jump performance and change-of-direction, studies reported significant gains. Both showed that improvements were maintained through eight-week detraining periods. Together, one Good-rated and one Fair-rated study support this outcome domain (moderate evidence; two Tier 1 studies).

For dynamic stability, meta-analysis received a Critically Low AMSTAR-2 rating (Table 1). The pooled SMD of 0.63 represents a moderate effect in magnitude. However, confidence in this estimate is limited by the study's methodological quality. Dynamic stability outcomes should therefore be rated as limited evidence, not moderate.

For agility, no superiority over traditional training was detected on standardised tests. This reflects measurement insensitivity rather than an absence of neuromuscular benefit. The RQ2 evidence base is stronger than that for RQ1. The stability evidence base is constrained by the low methodological quality of its sole available meta-analysis.

#### 7.1.3 Overall conclusion

**Figure 7:**
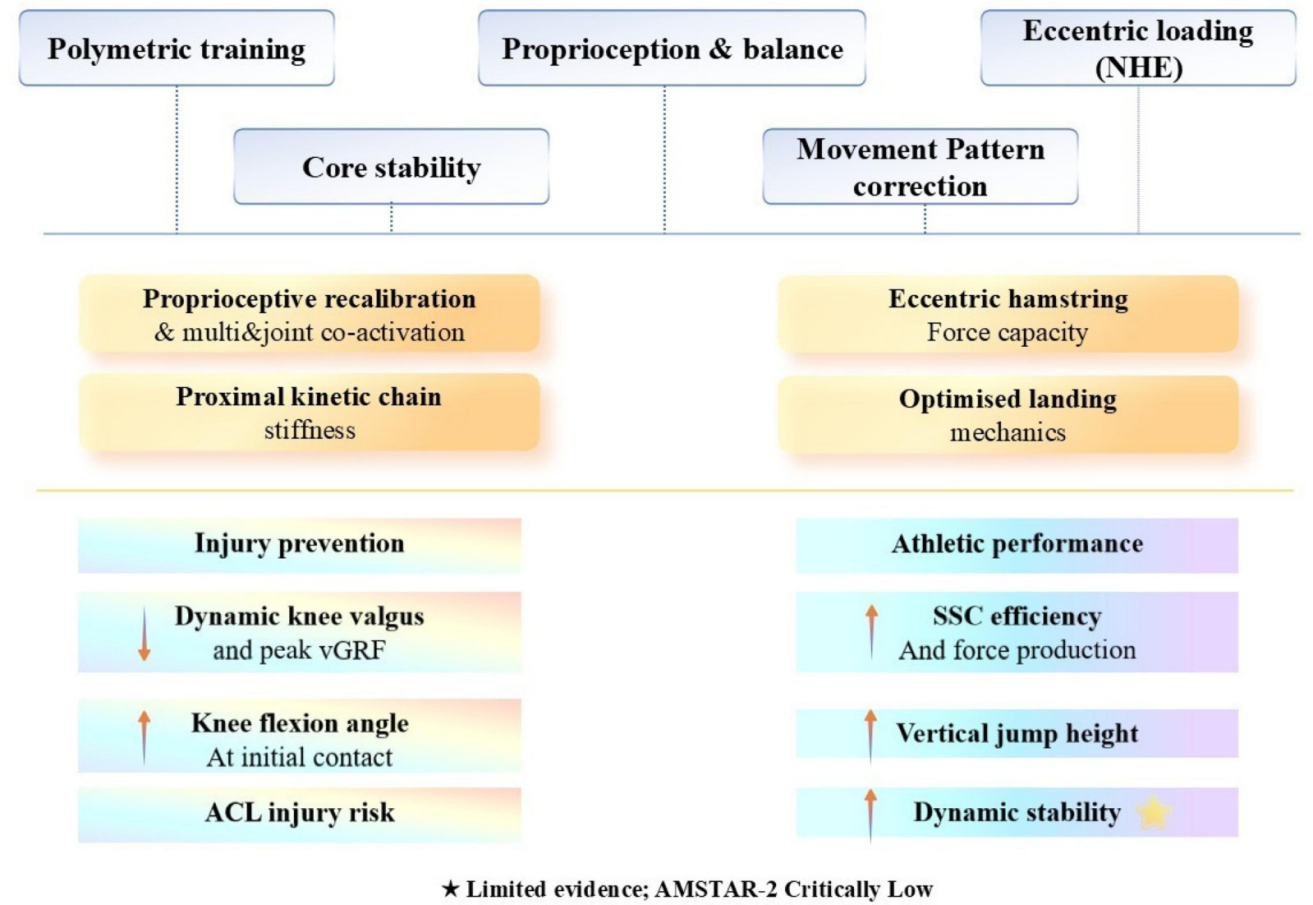
Common mechanistic pathways linking NMT to injury prevention and athletic performance in adolescent volleyball players.

Figure 7 illustrates the mechanistic structure underlying this dual benefit. Four shared neuromuscular adaptations sit at the centre of the diagram. Each is produced by the integrated NMT programme. Each simultaneously serves injury prevention and performance enhancement.

The first two adaptations address the distal control deficit. Both reduce the knee loading pattern that precedes ACL rupture. Both improve force generation and elastic energy storage at ground contact. The second two adaptations address the proximal control deficit. Both reduce the trunk displacement that amplifies knee abduction moment. Both extend the braking phase of the stretch-shortening cycle. The injury-protective and performance-enhancing effects are not separate outcomes of the same programme. They are the same physiological outputs expressed in different task contexts.

Four shared mechanistic adaptations link NMT to both injury prevention and performance enhancement. Each adaptation serves both functions simultaneously.

The first adaptation is proprioceptive recalibration and multi-joint co-activation. NMT shortens the neuromuscular feedback loop. It increases receptor sensitivity and pre-activation fidelity in the 40–50 ms window before foot contact. For injury prevention, this reduces dynamic knee valgus and the knee abduction moment that precedes ACL rupture. For performance, the same pre-activation capacity drives force production efficiency at ground contact. It directly determines vertical jump height and change-of-direction speed.

The second adaptation is eccentric hamstring force capacity. NHE training increases peak eccentric torque and shifts the torque-angle curve toward longer muscle lengths. For injury prevention, this reduces anterior tibial shear force at the critical landing-phase joint angles where ACL loading peaks. For performance, the same eccentric capacity prolongs the deceleration phase and improves the energy storage phase of the stretch-shortening cycle. This is a direct determinant of countermovement jump height.

The third adaptation is proximal kinetic chain stiffness. Core training increases trunk and hip neuromuscular co-activation. This prevents the lateral trunk displacement that amplifies knee abduction moment. For injury prevention, a stiffer trunk reduces the lever arm through which ground reaction force drives valgus loading. For performance, trunk stiffness provides the proximal platform from which hip and knee extensors can apply force most effectively during explosive jumping and landing tasks.

The fourth adaptation is optimized landing mechanics. Movement pattern correction training increases knee flexion and reduces peak vertical ground reaction force at initial contact. For injury prevention, deeper knee flexion reduces patellar tendon strain and ACL shear force. For performance, the same kinematic change extends the braking phase of the stretch-shortening cycle. A longer braking phase stores more elastic energy. More stored energy is available for the propulsive phase. This improves spike approach jump height directly.

These four pathways converge on a single conclusion. The biomechanical outputs that protect the knee joint are the same outputs that drive athletic performance. NMT does not trade injury safety for performance, or performance for safety. It develops the neuromuscular substrate from which both emerge.

The injury prevention and performance benefits of NMT share a common mechanistic foundation. Proprioceptive recalibration, multi-joint co-activation, eccentric hamstring strength, and proximal kinetic chain stiffness simultaneously reduce knee injury risk and enhance athletic performance. The injury-protective and performance-enhancing effects are mechanistically identical outputs expressed in different task contexts. For adolescent female volleyball players at or near PHV, a well-designed multimodal NMT program serves both objectives within a single training architecture. NMT framed exclusively as a prevention tool faces persistent uptake barriers in competitive settings. The dual-benefit framing supported by this review provides a practical rationale for embedding NMT into volleyball conditioning. Coaches need not choose between protecting and developing athletes. This conclusion rests substantially on extrapolated evidence and requires confirmation from volleyball-specific Tier 1 trials (moderate evidence).

### 7.2 Limitations of the current literature

Four principal limitations constrain confidence in applying findings to adolescent volleyball players. First, the evidence base is football-dominated: the only volleyball-specific NMT meta-analysis identified seven RCTs, forcing reliance on football and basketball data for mechanistic and dose-response conclusions (Tables 4, 5). Volleyball's defining features are not replicated in existing generic program. Second, prospective cohort data in adolescent volleyball are scarce; most studies used adult or mixed-age populations without maturity stratification. Third, considerable heterogeneity exists across studies in participant characteristics, outcome tools. As a structured narrative review, this study incorporates transparent search documentation and formal quality appraisal. However, it does not provide the exhaustive coverage or risk-of-bias pooling required of a systematic review. Conclusions should be interpreted within the constraints of narrative synthesis accordingly. Fourth, chronological age remains the dominant stratification variable despite biological maturity status being a stronger predictor of both injury risk and NMT responsiveness; field-applicable maturity methods are available but rarely applied. This omission prevents subgroup analysis by developmental stage and obscures the timing effects identified in Section 5.3 as critical determinants of NMT efficacy.

**Table 4:** Comparative NMT evidence density across pivoting and jumping sports.

Sport	No. of Eligible NMT RCTs	Representative Programme	Reported Injury Reduction	Adolescent-Specific Data	Volleyball-Specific Biomechanics Addressed
Football	Numerous (underpins international guidelines)	FIFA 11+ (16, 17)	Overall injury rate −39% (17)	Yes (FIFA 11+ Kids)	No — symmetric bilateral movements
Basketball	Growing (18)	Multicomponent neuromuscular warm-up (18)	Not quantified in this review	Limited	No
Handball	Growing (19)	Youth handball NMT protocols (19)	Not quantified in this review	Yes (youth female) (19)	No
Volleyball	7 eligible RCTs (20)	No validated sport-specific protocol	Stability improvement only: SMD = 0.63 (20)	Absent or age-unstratified (20)	No — asymmetric single-leg spike landing not replicated in any existing program

Numbers of eligible RCTs and injury-reduction effect sizes for basketball and handball are not formally quantified in this review; evidence density for these sports is characterised qualitatively based on cited reviews (18, 19). The volleyball row reflects the most comprehensive available volleyball-specific NMT meta-analysis (20). RCT counts and injury-reduction figures for football are based on FIFA 11 + evaluations (16, 17). No existing NMT programme has been designed to replicate asymmetric single-leg spike landings or high positional jump accumulation characteristic of volleyball. NMT, neuromuscular training; RCT, randomised controlled trial; SMD, standardised mean difference.

**Table 5:** PICOS framework defining eligibility criteria for tier 1 and tier 2 evidence inclusion.

Component	Tier 1 (Core evidence)	Tier 2 (Background evidence)
Population (P)	Adolescent athletes under 18 years engaged in volleyball training or competition	Adult volleyball players; athletes in other pivoting or jumping sports (football, basketball, handball)
Intervention (I)	NMT programmes incorporating one or more of: plyometric training, proprioceptive training, balance training, core stability training, eccentric resistance training, or structured landing mechanics correction	Same as Tier 1, or any NMT-related intervention relevant to mechanistic or epidemiological context
Comparison (C)	No-intervention control, traditional physical fitness training, or standard warm-up	Any comparator; no comparator required for epidemiological and biomechanical studies
Outcomes (O)	Primary: dynamic knee valgus, peak knee flexion angle at landing, vertical ground reaction force, neuromuscular control during sport-specific tasks. Secondary: vertical jump height, dynamic stability, agility	Mechanistic, epidemiological, or dose-response outcomes relevant to knee injury risk or NMT efficacy
Study design (S)	RCTs, quasi-RCTs, controlled trials with comparator; prospective longitudinal studies; systematic reviews and meta-analyses with adolescent volleyball players as primary population	RCTs, systematic reviews, meta-analyses, prospective cohort studies, cross-sectional biomechanical studies

RCT, randomised controlled trial. Tier 1 studies form the primary evidentiary basis for Sections 3–5. Tier 2 studies are used exclusively for mechanistic and epidemiological context and are explicitly identified throughout the text. Studies including both adolescent and adult athletes without age-stratified reporting are classified as Tier 2.

This extrapolation gap is most acute for the dose-response parameters in Section 6 and the biomechanical outcome claims in Section 4. The recommendations in this review should therefore be interpreted as evidence-informed rather than evidence-confirmed for this sport and age group. This distinction is clinically important. It defines the scope of the research agenda described in Section 7.3.

These four limitations reflect a single underlying gap: the near-complete absence of prospective RCT evidence in adolescent volleyball players as a primary study population. Closing this gap requires establishing a body of volleyball-specific adolescent NMT research that does not yet exist, as structured in the priorities outlined in Section 7.3.

### 7.3 Future research priorities

The limitations identified in Section 7.2 point directly to four research priorities. Addressing these gaps is necessary before high-confidence NMT recommendations can be made for the adolescent volleyball population.

Developing and validating volleyball-specific NMT program. No NMT program has been designed specifically for volleyball and validated in a prospective RCT. Current program are adapted from football and basketball protocols and do not replicate the movement patterns central to volleyball injury risk. Asymmetric single-leg spike landings, unanticipated block jumps, and positional jump accumulation profiles are absent from existing generic program. Future trials should develop program targeting these sport-specific patterns directly. Outside hitters and middle blockers carry the greatest injury exposure and should be studied as distinct subgroups. Program validation should include both biomechanical landing outcomes and prospective injury incidence as primary endpoints.

Maturity-stratified intervention studies. Biological maturity status predicts both injury risk and NMT responsiveness more precisely than chronological age. Yet no published volleyball NMT trial has stratified participants by maturity status. Future studies should classify participants as pre-PHV, circa-PHV, or post-PHV at enrolment using validated field methods, including maturity offset, percentage of predicted adult height, and the Khamis–Roche equation. This stratification would allow direct comparison of NMT efficacy across maturation stages and test whether the circa-PHV window produces greater protection than interventions introduced at other stages. Without it, the developmental timing argument central to this review cannot be directly examined.

The role of cognitive-fatigue interaction in injury mechanism. Standard laboratory fatigue protocols produce inconsistent effects on landing biomechanics. They isolate physiological fatigue from the cognitive demands present in real match conditions. In volleyball, spike landings follow high-intensity rallies and require simultaneous tactical decision-making. Physical fatigue and concurrent cognitive load interact to degrade knee biomechanics more than either factor alone. Future studies should use dual-task paradigms combining physiological fatigue induction with concurrent visuomotor or decision-making demands. Such designs would more accurately replicate the neuromuscular state preceding real-world ACL injury. They would also allow testing of whether NMT incorporating fatigue-state training produces greater protection than NMT delivered under rested conditions.

Standardization of outcome measures. Dynamic stability, landing mechanics, and performance outcomes are currently measured using different tools across volleyball NMT studies. This prevents meaningful meta-analytic pooling and cross-study comparison. Future trials should adopt a standardized core outcome set including at minimum: peak knee flexion angle and knee abduction moment during sport-specific single-leg landing tasks, Y Balance Test composite score, countermovement jump height, and a reactive agility measure capturing unanticipated directional response. Maturity assessment should be reported alongside chronological age as a standard requirement.
