# Supplementary material for: Dual benefits of neuromuscular training in adolescent volleyball players: knee injury prevention and athletic performance share a common mechanistic foundation: a structured narrative review
Source: Front Sports Act Living. 2026 Jun 24;8:1849255. doi: 10.3389/fspor.2026.1849255 (PMC13341738; doi:10.3389/fspor.2026.1849255)
Supplement: Supplementary file 1 [file Table1.docx]

**The note of Table 1:** Numbers of eligible RCTs and injury-reduction effect sizes for basketball and handball are not formally quantified in this review; evidence density for these sports is characterised qualitatively based on cited reviews [19, 20]. The volleyball row reflects the most comprehensive available volleyball-specific NMT meta-analysis [21]. RCT counts and injury-reduction figures for football are based on FIFA 11+ evaluations [17, 18]. No existing NMT programme has been designed to replicate asymmetric single-leg spike landings or high positional jump accumulation characteristic of volleyball. NMT = neuromuscular training; RCT = randomised controlled trial; SMD = standardised mean difference.

**The note of Table 2:** Note. Tier 1 classification requires adolescent volleyball players as the primary study population with reported NMT biomechanical or performance outcomes (Section 2.2). Age stratification by maturity status was not reported in any included study. Yang et al. [21] is a meta-analysis of seven RCTs; individual RCT-level characteristics are not fully extractable from the source publication. *PEDro scores to be completed by authors following full-text review prior to manuscript submission. CMJ = countermovement jump; COD = change of direction; NMT = neuromuscular training; RCT = randomised controlled trial; SMD = standardised mean difference.

**The note of Table 3:** RCT = randomised controlled trial. Tier 1 studies form the primary evidentiary basis for Sections 3–5. Tier 2 studies are used exclusively for mechanistic and epidemiological context and are explicitly identified throughout the text. Studies including both adolescent and adult athletes without age-stratified reporting are classified as Tier 2.

**The notes of Table 4:** Full item-level ratings for PEDro and AMSTAR-2 are provided in Tables S1 and S2 of the Supporting Information. PEDro quality grades: Excellent = 9–10; Good = 6–8; Fair = 4–5; Poor ≤ 3 [28]. AMSTAR-2 confidence ratings: High, Moderate, Low, Critically Low [29]. Quality ratings were not used as exclusion criteria. All three Tier 1 studies are retained in the evidence synthesis regardless of quality grade.

**Notes of Table S1 and Table S2:** PEDro = Physiotherapy Evidence Database scale. Scored items: 2–11 (maximum score 10); item 1 specifies eligibility criteria and is not included in the score. Quality grades: Excellent = 9–10; Good = 6–8; Fair = 4–5; Poor = ≤3 (Maher et al. [24]). Y = criterion met; N = criterion not met (by design — blinding not achievable in exercise intervention studies); further full-text verification required. Cells marked with ★ are critical PEDro domains. Nunes et al. [26] = cluster-RCT; Gavala et al. [27] = controlled trial with comparator group.

AMSTAR-2 = A Measurement Tool to Assess Systematic Reviews [29]. Critical domains (★): items 2, 4, 7, 9, 11, 13, and 15. Overall confidence ratings: High (no or one non-critical weakness); Moderate (more than one non-critical weakness); Low (one critical flaw); Critically Low (more than one critical flaw). Y = criterion confirmed met; N = criterion confirmed not met; ? = unable to determine from available published information — full-text review required prior to final submission. The overall Critically Low rating reflects four unresolved or unmet critical domains: item 7 (?), item 9 (?), item 13 (?), and item 15 (N). Quality ratings were not used as exclusion criteria. Yang et al. [21] is retained in the synthesis regardless of this rating.

**Table S1 Randomised and Quasi-Randomised Controlled Trials — PEDro Scale**

| Item | Pedro criterion and rationale | Nunes et al. 2021 [26] | Gavala et al. 2023 [27] |
| --- | --- | --- | --- |
| 1 | Eligibility criteria specified. Population, intervention, and outcomes defined a priori. Item 1 is not included in the total score. | Y | Y |
| 2 | Random allocation. Nunes [26]: cluster-RCT; participants cluster-randomised into intervention (INT) and control (CON) groups prior to baseline assessment. Gavala [27]: participants divided into intervention group (INTG) and control group (CG) by the investigator without a reported randomisation procedure; design classified as a controlled trial with a comparator group. | Y | N |
| 3 | Concealed allocation. Neither study reported use of a concealed allocation mechanism (e.g., sealed envelopes, central allocation). The cluster-RCT design of [26] limits allocation concealment feasibility at the participant level; the non-randomised design of [27] precludes this criterion entirely. | N | N |
| 4 | Baseline comparability. Both studies reported baseline characteristics (age, body mass, height, and performance outcomes) for each group separately and confirmed no significant between-group differences at baseline. | Y | Y |
| 5 | Blinding of participants. Not achievable by design; participants cannot be blinded to their training condition in exercise intervention studies. Criterion not met in either study. | N | N |
| 6 | Blinding of therapists. Not achievable by design; coaches and trainers delivering the NMT protocols cannot be blinded to group assignment. Criterion not met in either study. | N | N |
| 7 | Blinding of assessors. Neither study reported blinded outcome assessment. Primary outcomes (countermovement jump height, spike jump, agility) were measured by study personnel who were not reported to be blinded to group allocation. | N | N |
| 8 | Completeness of follow-up (≥85%). Nunes [26]: n = 32 completed all assessment time points (weeks 0, 6, 12, and 20); no participant attrition reported. Gavala [27]: n = 61 completed the 12-week intervention and 8-week detraining follow-up; no dropouts reported. Both exceed the 85% threshold. | Y | Y |
| 9 | Intention-to-treat analysis. Both studies analysed all enrolled participants at each time point without post-allocation exclusions, consistent with ITT principles. No imputation procedures were required given complete follow-up in both trials. | Y | Y |
| 10 | Between-group statistical comparisons. Both studies reported group × time interaction statistics. Nunes [26]: repeated-measures ANOVA with Bonferroni post hoc correction. Gavala [27]: two-way repeated-measures ANOVA with significant group × time interaction (p < 0.001) reported for all power performance outcomes. | Y | Y |
| 11 | Point estimates and variability reported. Both studies reported means, standard deviations, p-values, and effect sizes (Cohen's d or partial η²) for all primary outcome measures. | Y | Y |
| - | Total score (items 2–11) / 10  Items 5 and 6 are structurally unattainable in exercise intervention studies (N by design, not by methodological deficiency). | 6/10 | 5/10 |
| - | Quality grade | Good | Fair |

(Table note is supplied in the Supporting information).

**Table S2 Systematic Reviews and Meta-Analyses — AMSTAR-2**

| Item | ★ | AMSTAR-2 Domain | Yang et al. 2026 [17] |
| --- | --- | --- | --- |
| 2 | ★ | PICO question and inclusion criteria specified. Research questions and inclusion criteria defined a priori. Population (volleyball athletes), intervention (NMT), comparator, and outcomes (stability, performance) are clearly stated. | Y |
| 3 |  | Prospective protocol registered. Registration in a prospective registry (e.g., PROSPERO) prior to data collection. Registration status could not be confirmed from available published information. | ? |
| 4 | ★ | Comprehensive literature search. Search conducted in at least two databases, including grey literature. Six databases searched: PubMed, SPORTDiscus, Web of Science, Scopus, Cochrane, EMBASE. | Y |
| 5 |  | Study selection performed in duplicate. At least two independent reviewers selected studies. Could not be confirmed from available published information. | ? |
| 6 |  | Data extraction performed in duplicate. At least two independent reviewers extracted data. Could not be confirmed from available published information. | ? |
| 7 | ★ | Excluded studies listed with reasons. A list of excluded studies with justification provided. PRISMA flow diagram present; however, a full exclusion list with reasons could not be confirmed from available published information. | ? |
| 8 |  | Included studies described in adequate detail. Sufficient detail on populations, interventions, and outcomes reported. Study characteristics are summarized in Table 2 of this review. | Y |
| 9 | ★ | Risk of bias assessed for individual studies. A validated tool (e.g., Cochrane RoB, PEDro) used for each included study. Could not be confirmed from available published information. | ? |
| 10 |  | Funding sources of included studies reported. Sources of funding for each included primary study reported. Not reported in available published information. | N |
| 11 | ★ | Appropriate statistical methods used. Appropriate pooling method used; heterogeneity assessed. SMD with 95% CI reported; I² = 67.5% for dynamic stability; sex-stratified subgroup analyses conducted. | Y |
| 12 |  | Impact of RoB on pooled estimates considered. Sensitivity analyses conducted excluding high-risk-of-bias studies. Could not be confirmed from available published information. | ? |
| 13 | ★ | Results of individual studies explained and discussed. Interpretation of results accounts for risk of bias. Could not be confirmed from available published information. | ? |
| 14 |  | Heterogeneity discussed and explained. Sources of heterogeneity identified and discussed. I² = 67.5% reported; sex-stratified subgroup analyses conducted to explore variation. | Y |
| 15 |  | Publication bias assessed and discussed. Methods such as funnel plot or Egger test reported. Not reported in available published information. | N |
| 16 |  | Conflicts of interest reported. Potential conflicts of interest of review authors and included studies disclosed. Could not be confirmed from available published information. | ? |
| — | — | Overall AMSTAR-2 Confidence Rating (High \| Moderate \| Low \| Critically Low) | low |
